# Understanding the Chemical Degradation of Ti_3_C_2_T_*x*_ MXene Dispersions: A Chronological Analysis

**DOI:** 10.1002/smsc.202400150

**Published:** 2024-06-25

**Authors:** Kevinilo P. Marquez, Kim Marie D. Sisican, Rochelle P. Ibabao, Roy Alvin J. Malenab, Mia Angela N. Judicpa, Luke Henderson, Jizhen Zhang, Ken Aldren S. Usman, Joselito M. Razal

**Affiliations:** ^1^ Institute for Frontier Materials Deakin University Geelong VIC 3216 Australia; ^2^ Institute of Chemistry University of the Philippines‐Los Baños Laguna 4031 Philippines; ^3^ Manufacturing Commonwealth Scientific and Industrial Research Organization (CSIRO) Waurn Ponds VIC 3216 Australia

**Keywords:** MXenes, oxidation, solution‐based processing, timeline

## Abstract

Titanium carbide (Ti_3_C_2_T_*x*_) MXene has attracted significant attention due to its exceptional properties and versatile solution processibility. However, MXene dispersions are prone to various degradation processes, leading to the formation of byproducts that negatively affect its morphological, electrical, and mechanical properties. Through the years, several methods have been developed to mitigate MXene degradation; however, divergent viewpoints on the understanding of degradation mechanisms are prevalent, hindering the development of versatile strategies in producing environmentally stable MXene dispersions. This review provides a chronological analysis of the research efforts aimed at unraveling the underlying mechanisms of MXene degradation and highlights strategies for circumventing this process. This review discusses apparent inconsistencies in experimental findings and theoretical models. These discrepancies prompt further investigation for a clearer understanding of the degradation process in MXene. This narrative allows readers to follow the evolution of dominant theories and disputes and to ultimately stimulate further investigation, aiming for a better understanding of this process. It is anticipated that identifying the fundamental factors affecting the oxidation of MXene dispersions will enable their full‐scale processing into higher‐order structures and practical devices with greater longevity and long‐term performance.

## Graphical abstract

This review provides a chronological overview of the research efforts aimed at unraveling the underlying mechanisms of MXene oxidation. Additionally, it enables readers to track the evolution of primary theories and disagreements, contributing to the current state of ambiguity and discord within literature.

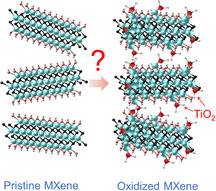

## 1 Introduction

The discovery of two‐dimensional (2D) transition metal carbides and carbonitrides in 2011^[^ ^1^ ^]^ led to a family of materials referred to as “MXenes” (M_*n*+1_X_*n*_T_*x*_, *n* = 1–4, where M = transition metal, X = carbon and/or nitrogen, and T = —O—, =O, —OH, —F, —Cl). Characterized by a highly processable two‐dimensional layered structure and noteworthy physicochemical properties, MXenes have captured significant interest across multiple application domains.^[^ ^2^, ^3^, ^4^, ^5^, ^6^, ^7^, ^8^ ^]^ To date, more than 50 stoichiometric MXene compositions have been experimentally reported, and several other unique metal carbide/carbonitride combinations have been theoretically predicted.^[^ ^9^, ^10^ ^]^ This review focuses on the widely studied Ti_3_C_2_T_*x*_ MXene (herein referred to as MXene for simplicity), distinguished among other 2D nanomaterials by its exceptional electrical conductivity (≈20 000–24 000 S cm^−1^)^[^ ^11^, ^12^ ^]^ and electrochemical capacitance (>2800 F cm^−3^).^[^ ^13^ ^]^

MXenes are commonly prepared from transition metal carbide/nitride precursors, known as MAX phases (M_*n*+1_AX_*n*_ where M = metal; X = C or N; A = elements in Groups 11–14, most commonly Al) through various top‐down A‐layer etching methods resulting in single‐ and multilayered sheets.^[^ ^11^, ^14^, ^15^, ^16^ ^]^ The removed A‐layer is immediately replaced by groups derived from the components present in the etchant, forming the surface terminations (T), which typically comprises of electron‐rich (contains nonbonded but active valence electrons, e.g., N, O, F, S) atoms or groups, and capable of hydrogen bonding. Fluorine (—F) and oxygen species (—O—, =O, —OH) are prevalent for HF‐etched MXenes but may vary between direct and in situ etching methods.^[^ ^17^, ^18^ ^]^ Typical HF‐based methods (e.g., aqueous HF, HCl—HF) usually produce MXenes with relatively higher —Cl and —F terminations while the in situ HF methods produce relatively higher —O—, =O, and —OH terminations. Although less common, fluorine‐free etching can also be employed to introduce other T species (e.g., —Cl, —Br, nitrogen groups) and intercalating cations (e.g., K^+^, Na^+^, Al^3+^, N(C_4_H_9_)_4_^+^). These methods include the use of quaternary ammonium hydroxides (TMAOH, TBAOH),^[^ ^19^ ^]^ molten salts (ZnCl_2_, LiCl),^[^ ^20^ ^]^ and mechanochemical exfoliation.^[^ ^21^ ^]^

MXene sheets acquired using the more scalable and reproducible fluoride‐based routes (HF, HCl‐LiF, and/or HCl/HF), typically possess highly active surface groups, giving them high dispersibility in water (*ζ*‐potential of −30 to −60 mV) and various polar organic solvents.^[^ ^22^ ^]^ This facilitates easy solution processing without the need for stabilizers or surfactants and has led to numerous investigations utilizing diverse solution‐based techniques, transforming MXene into versatile structures such as films,^[^ ^23^, ^24^ ^]^ fibers,^[^ ^25^ ^]^ and aerogels.^[^ ^26^, ^27^ ^]^ This assortment of MXene structures and architectures can be adapted for many applications, including but not limited to energy storage,^[^ ^23^, ^28^ ^]^ energy harvesting,^[^ ^29^ ^]^ sensors,^[^ ^30^ ^]^ and electromagnetic interference shielding.^[^ ^31^ ^]^

Comprehensive investigations into the properties of MXene dispersions such as viscosity and viscoelasticity^[^ ^6^, ^32^ ^]^ and the discovery of their liquid crystal phase^[^ ^6^, ^25^, ^28^ ^]^ have enabled the fabrication of multifunctional macrostructures. However, the susceptibility of these dispersions to degradation at room temperature (RT) affects their scalability in MXene‐based devices.^[^ ^33^, ^34^, ^35^ ^]^ This leads to the deformation of the original sheet morphology and growth of unwanted oxide phases, transforming the sheets into a mixture of titanium‐ and carbon‐based degradation products (TiO_2_, CO_2_, CO, and CH_4_),^[^ ^6^, ^32^, ^36^ ^]^ consequently diminishing their processibility into functional architectures. The morphological changes on the surface and edges of the flakes brought by oxidative degradation also result in a marked decrease in shelf life and key properties like conductivity. This loss of conductivity in MXene limits its potential in applications where high electrical conductivity provides an advantage, hence, understanding and mitigating the effects of oxidative degradation has become a critical point for exploration in this field.

Several mechanisms for the degradation process have been postulated since the first study by Zhang et al.^[^ ^33^ ^]^ However, these studies have yet to arrive at a consensus regarding the actual mechanism. Many studies have confirmed the transformation of dispersed MXene to TiO_2_, amorphous carbon, as well as gaseous products such as CO, CO_2_, and including CH_4_, which is a by‐product that was not previously known to form by oxidation. However, there are contradictions in identifying which agents or factors contribute significantly to the degradation process. For example, Xia et al. provided a mechanism based on the susceptibility of Ti vacancies to dissolved oxygen.^[^ ^37^ ^]^ In contrast, Doo et al. proposed an acid‐catalyzed oxidation reaction via the hydroxyl groups on the surface of MXene.^[^ ^38^ ^]^ Ultimately, these contrasting beliefs hinder the development of a versatile method for preserving MXene dispersions, even while several mitigation methods have already reported.^[^ ^39^, ^40^, ^41^ ^]^ This review offers a chronological overview of pivotal studies that have contributed to understanding the mechanisms involved in the degradation of MXene in dispersions. Although several outstanding reviews on MXene oxidation exist,^[^ ^34^, ^35^, ^42^ ^]^ this narrative focuses on the evolution of prevailing and contradictory conclusions that have contributed to the current discord in the literature. We believe that unraveling the root of these debates can aid in identifying the fundamental factors influencing MXene oxidation. This review also includes how mitigation strategies were developed to prolong the shelf life of MXene dispersions, in relation to the existing knowledge on the degradation mechanisms. Finally, we examine potential opportunities arising from the discussed challenges and anticipated obstacles in studying MXene oxidation. Future research directions that pay particular attention to other critical factors and parameters that could enhance the understanding of MXene oxidation are derived. Overall, we envision that the narrative offered by this review will allow researchers to develop more efficient and versatile strategies in addressing MXene oxidation and producing stabilized MXene to expand its range of applications.

## 2 Timeline of Critical Research on Ti_3_C_2_T_*x*_ Degradation

**Figure 1:**
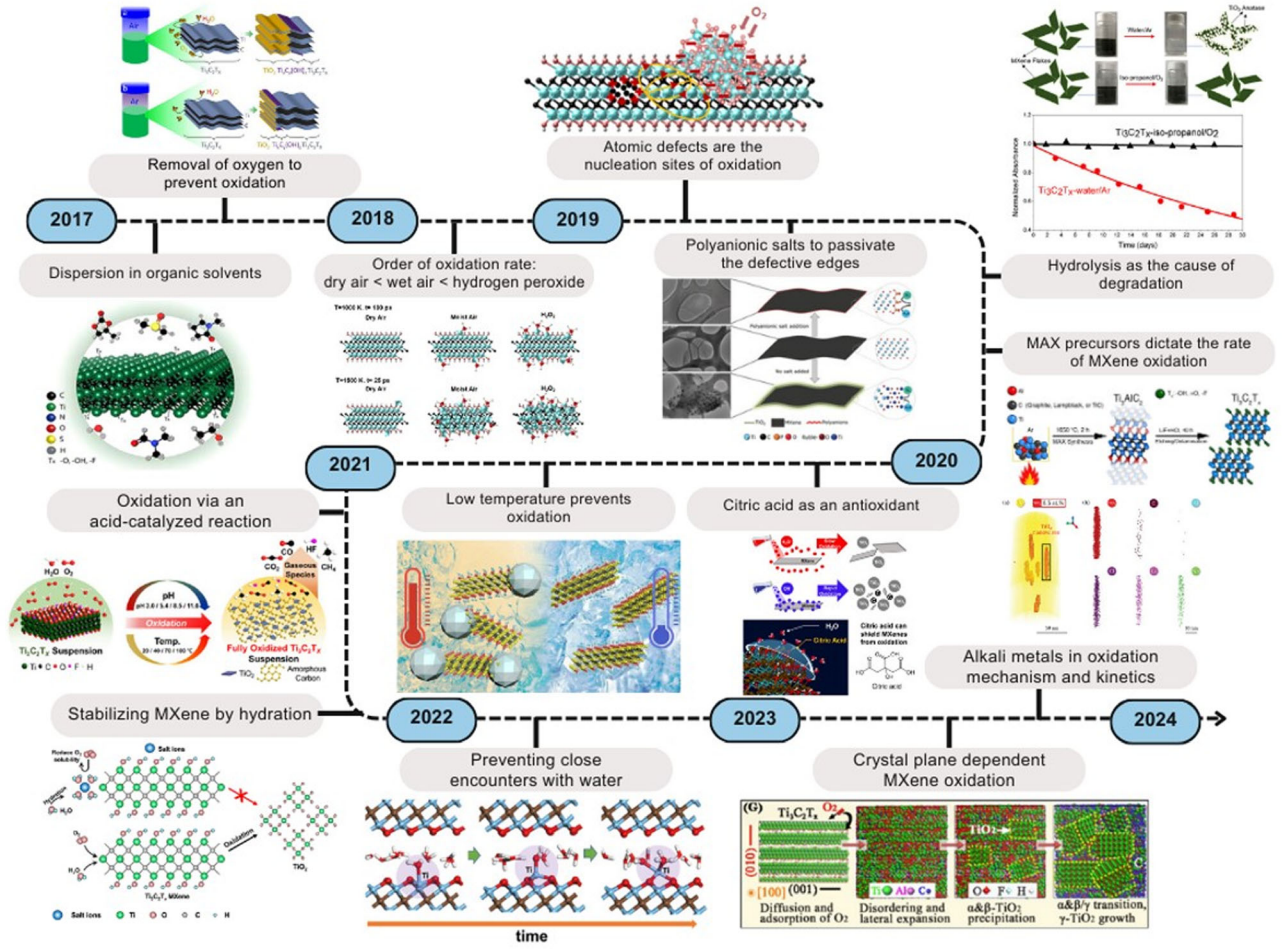
Timeline of studies exploring causes and mechanisms of Ti_3_C_2_T_*x*_ degradation. Adapted with permission.^[^ ^22^ ^]^ Copyright 2017, American Chemical Society.^[^ ^33^ ^]^ Copyright 2017, American Chemical Society.^[^ ^51^ ^]^ Copyright 2017, American Chemical Society.^[^ ^37^ ^]^ Copyright 2018, Royal Society of Chemistry.^[^ ^47^ ^]^ Copyright 2019, American Chemical Society.^[^ ^57^ ^]^ Copyright 2019, American Chemical Society.^[^ ^44^ ^]^ Copyright 2019, American Chemical Society.^[^ ^58^ ^]^ Copyright 2019, American Chemical Society.^[^ ^40^ ^]^ Copyright 2020, American Chemical Society.^[^ ^38^ ^]^ Copyright 2021, American Chemical Society.^[^ ^64^ ^]^ Copyright 2021, Wiley‐VCH.^[^ ^53^ ^]^ Copyright 2022, American Chemical Society.^[^ ^66^ ^]^ Copyright 2024, Wiley‐VCH.^[^ ^67^ ^]^ Copyright 2024, Wiley‐VCH.

Understanding the degradation processes of MXene plays a vital role in the development of strategies that extend the shelf life of their dispersions. The current literature presents numerous morphological and chemical factors driving the degradation process. This section provides a chronological overview of studies that have contributed to our comprehension of MXene oxidation over time (**Figure** **1**). Also, an extensive summary of the literature is presented in **Table** **1**. The goal of this section is to elucidate prevailing viewpoints and identify conflicting ideas in the literature. Each subsection highlights strategies formulated to address the limited stability of MXene dispersions based on available information at the time.

**Table 1:** Representative studies exploring causes and mechanisms of Ti_3_C_2_T_*x*_ oxidation from 2017 to 2024.

	Year	Type	Oxidants	Etching method	Experimental factors				Conditions (pH, Temperature, solvent system)	Time before completec) degradation, days	Mitigation strategies	Reference
					MAX	Environment		Additives				
					MXene	Chemical	Physical					
1	2017	a)	O_2_	MILD	MXene flake size	–	Temperature	–	Air, 23 °C (RT), water as solvent	60	Storage of MXene solutions in Ar‐filled bottles at 5 °C.	[33]
2	2017	a)	O_2_, H_2_O	LiF–HCl (clay) 50% HF (powder)	–	Solvent	–	–	Air, Polar organic solvent systems	40	Dispersion in polar organic solvent (N,N‐dimethylformamide, N‐methyl‐2‐pyrrolidone, dimethyl sulfoxide, propylene carbonate, and ethanol).	[22]
3	2019	a)	O_2_, H_2_O	LiF–HCl (12 m)	–	–	–	–	Air, RT, water as solvent	20	Synthesize high‐quality MXenes. Minimize basal plane and edge defects.	[37]
4	2019	a)	O_2_, H_2_O	LiF–HCl (12 m)	MXene type: Ti_3_C_2_, V_2_C	Atmosphere, Polyanions	–	–	Air, RT, water as solvent	30	Edge‐cap positively charged MXene by the polyanions such as polyphosphate salts.	[57]
5	2019	a)	H_2_O	LiF–HCl (6 m)	MXene type: Ti_3_C_2_, Ti_2_C	Solvent, atmosphere	–	–	RT, water, and iso‐propanol as solvents	41	Protect MXene from exposure to water (use organic solvents) rather than oxygen.	[44]
6	2019	a)	O_2_, H_2_O	MILD	–	Solvent, atmosphere	Temperature	–	Air, temperature of 5 °C, −18 °C, −80 °C, water and ethanol as solvent	70	MXene is kept stable for more than 39 weeks when stored at −80 °C, MXene dispersed in ethanol can also delay degradation.	[59]
7	2019	a)	O_2_, H_2_O	LiF–HCl (6 m)	–	–	Temperature, UV light	Polymer matrices	Air, RT, liquid (water, acetone, acetonitrile), solid (ice and polymer) media	64	MXene can be preserved in ice (and some organic solvents) or freeze‐dried to form a re‐dispersed powder.	[73]
8	2019	a)	O_2_, H_2_O	LiF–HCl (12 m)	MAX precursors: graphite, carbon lampblack, TiC	–	–	–	RT, water as solvent	12	TiC‐produced MXene showed the highest stability; synthesis of MAX precursor affects MXene properties.	[58]
9	2019	a)	O_2_, H_2_O	MILD	–	Solvent	–	–	RT, deaerated water, and organic solvent systems	28	Deaerating the dispersion with Ar (removal of oxygen) or using organic solvents (removal of water) through SE method.	[43]
10	2020	a), b)	O_2_, H_2_O, ·OH	LiF–HCl (12 m)	MXene type and conc.	pH	–	Citric acid	RT, pH 2.0–10.0, water as solvent	40	MXene dispersions should be prepared in high concentration, acidic pH, and citric acid.	[60]
11	2020	a)	H_2_O	MILD	MXene type	–	Temperature	–	70 °C, water as solvent	10	–	[36]
12	2020	a)	O_2_, H_2_O	MILD	–	–	Temperature	–	1 mL air, closed, −20 °C, 4 °C, and RT, water as solvent	650	Freezing aqueous MXene dispersions at –20 °C.	[40]
13	2021	a)	O_2_	LiF–HCl (12 m)	Modified MAX phase	–	–	–	Closed, RT, water as solvent	300	Excess aluminum in MAX phase synthesis produces MXenes of higher quality.	[12]
14	2021	a)	O_2_, H_2_O	MILD	–	pH	Temperature	–	pH 3.0–11.0, 20–100 °C, water as solvent	60	–	[38]
15	2021	a)	O_2_, H_2_O	LiF–HCl (6 m)	–	–	–	Inorganic salts	Saturated salt solution as solvent	30	Hydration effect of inorganic salts; rinse and restore MXene.	[64]
16	2022	b)	H_2_O	LiF–HCl (6 m)	–	–	–	–	Not applicable	–	Control the exposure to water.	[53]
17	2022	a)	H_2_O	LiF–HCl (6 m)	–	–	–	Antioxidants: a‐hydroxy acids, polycarboxylic acids, phenolic compounds	α‐hydroxyacids, polycarboxylic acid, phenolic compounds as solvents, closed, ambient conditions	14	–	[39]
18	2023	a), b)	O_2_, H_2_O	LiF–HCl (12 m)	–	–	–	SDS	Air, RT, SDS solution as solvent	213	Addition of 1.5 mg mL^−1^ SDS.	[65]
19	2023	a)	–	NaOH (27.5 m) @ 270 °C, 72 h	Halogen‐free MXene	–	–	–	NaOH solution as solvent	28	NaOH‐based hydrothermal etching of MXene.	[71]
20	2023	a)	O_2_, ·OH	LiF–HCl (12M)	–	–	–	Imidazolium salts	Water as solvent	30	–	[76]
21	2024	a), b)	O_2_	40% HF	–	–	–	–	Air, RT, water as solvent	In situ analysis	–	[67]
22	2024	a), b)	O_2_, H_2_O	LiF–HCl (12M)	–	–	–	Tris‐HCl	pH 7.0, RT, Tris HCl solution as solvent	150	Addition of Tris‐HCl for pH control.	[77]

a) experimental studies;

b) computational studies;

c) completeness is dependent on the parameters set by the authors.

Since their discovery, MXenes have shown immense potential as active components for flexible and environmentally robust wearable devices. From this, two major research directions emerged—one is aimed at expanding the number of available techniques for synthesizing^[^ ^15^, ^16^ ^]^ and processing^[^ ^22^, ^32^, ^43^ ^]^ of MXenes to increase its overall manufacturability, while the other is focused on recognizing the key factors^[^ ^14^, ^33^ ^]^ and mechanistic processes^[^ ^36^, ^44^, ^45^, ^46^ ^]^ that govern the degradation of MXene. The latter is dedicated to the mitigation and potential prevention of MXene degradation, through either modification in synthesis and processing conditions (temperature, pH) or by the addition of stabilizing agents (antioxidants, capping agents). MXene degradation research further branches out onto investigating structural (e.g., presence of defects, nature of surface terminations and flake edges) and environmental (e.g., temperature, UV radiation, pH, solvent system) factors. Mechanistic processes, such as redox (electron‐transfer reactions), hydrolysis (water‐induced), and catalytic factors (from agents that hasten the rate of degradation), are proposed to explain the effects and predict the reactivity of MXenes. These strategies are synergistic, often solving the limitations raised by another approach (e.g., use of organic solvents, edge capping, freezing, etc.). However, the overall development of standardized methods is severely limited by the differences in the quality of starting materials (MAX phase) and MXene synthesis methods. Factors also include pre‐ and postsynthetic treatments, which adds to the variations that need to be considered when drawing comparisons between studies.

### 2.1 (2017–2018) Early Studies on Oxidation—Role of Dissolved Oxygen and Solvent

#### 2.1.1 Early Observations on Conversion of MXene to TiO_*2*_

**Figure 2:**
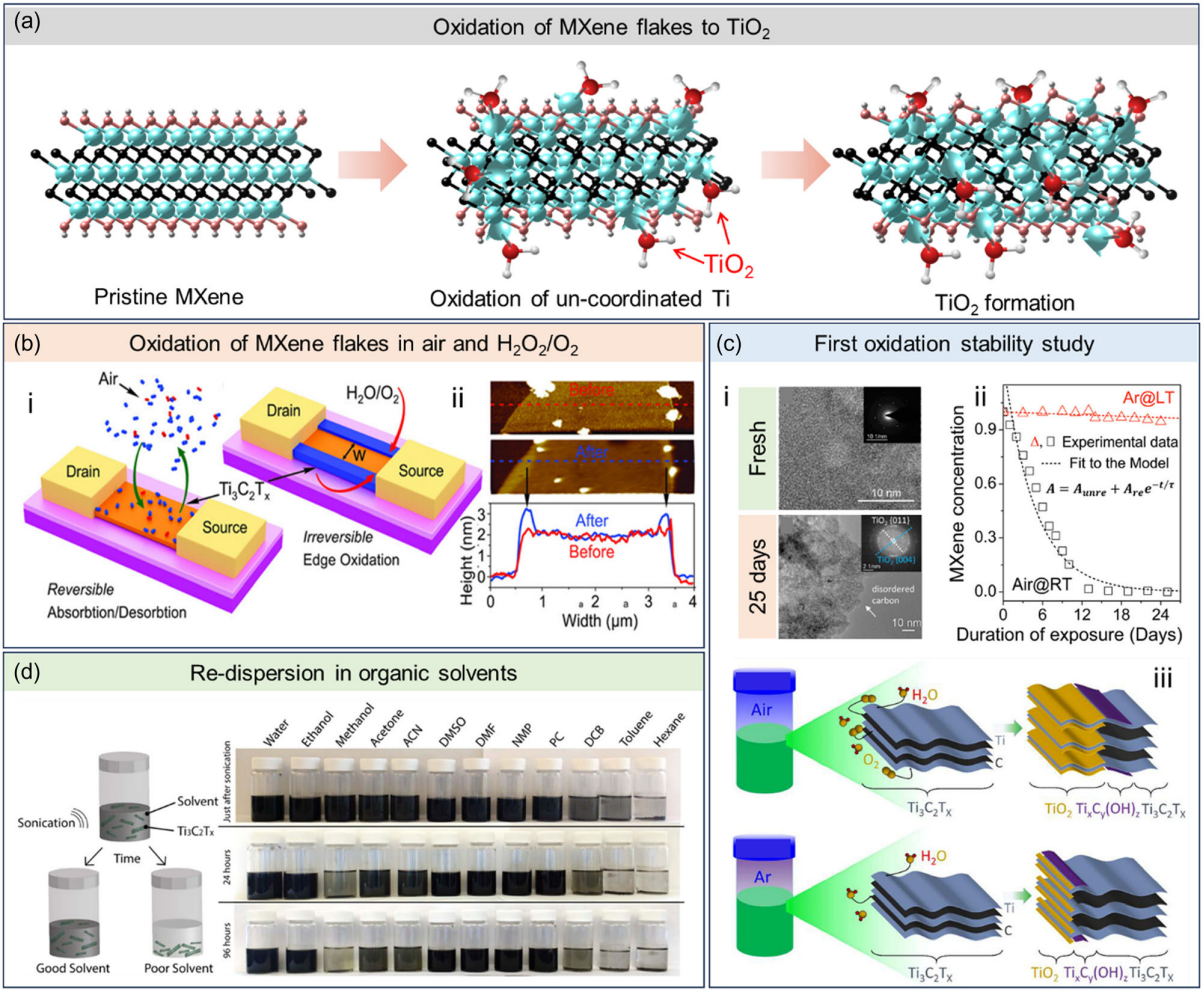
a) Partial structural transformation of MXene (Ti_3_C_2_T_*x*_) to TiO_2_, as first observed by Mashtalir et al. in 2014.^[^ ^47^ ^]^ b) Oxidation of MXene sheets to a relatively less‐conductive TiO_2_ phase. (i) Distribution of TiO_2_ when exposed under humid air and H_2_O_2_/O_2_. (ii) AFM image of fresh and oxidized MXene showing an increase in height profile for the edge of each sheet. Adapted with permission.^[^ ^50^ ^]^ Copyright 2016, Wiley‐VCH. c) First oxidation stability study by Zhang et al. (i) TEM images of MXene stored in air at RT. (ii) Plot showing that complete decomposition of MXene in air at RT (Air@RT) happens after 30 days but was prevented by storing the dispersion in Ar under low temperature (AR@LT). (iii) Schematics of degradation of aqueous MXene solution in Air@RT and Ar@LT. Adapted with permission.^[^ ^33^ ^]^ Copyright 2017, American Chemical Society. d) 50% HF‐etched MXene in 12 different solvents to identify which system is capable of dispersing and subsequently storing MXenes over a long period of time. Adapted with permission.^[^ ^22^ ^]^ Copyright 2017, American Chemical Society.

The conversion of delaminated MXene to nanoscale anatase TiO_2_ was first reported in a study by Mashtalir et al.^[^ ^47^ ^]^ where they monitored the structural changes of MXene dispersed in aqueous media. This study was the first to suggest that dissolved oxygen molecules in an aqueous solution oxidize MXene upon their adsorption onto undercoordinated Ti atoms, which are more susceptible to attack from electron‐rich species due to the presence of vacant bonding sites (**Figure** **2**a). However, by that time, the mechanism of this process was deciphered only theoretically for a different 2D titanium carbide (Ti_2_CT_*x*_).^[^ ^48^ ^]^ With the aid of density functional theory (DFT) and molecular dynamics (MD) simulations, it was predicted that the unsaturated Ti *3d* orbitals of the pristine monolayer Ti_2_CT_*x*_ surface react with the O_2_ molecule during the initial stage of the oxidation process. This results in fast O_2_ dissociation and facilitates the diffusion of the dissociated O atoms throughout the structure. The insight that oxidation occurs between atmospheric O_2_ and uncoordinated Ti in MXene was derived from the oxidation mechanism experimentally observed on bulk anatase. At that time, such a process occurring in undercoordinated Ti sites in MXene had not yet been observed experimentally. This insight, however, was further put forward by another study^[^ ^49^ ^]^ aimed at controlling the growth of anatase formed on the surface while keeping the structure of MXene pristine underneath.

A study by Lipatov et al. investigated the quality, electronic properties, and environmental stability of monolayer Ti_3_C_2_T_*x*_ MXene sheets. The study demonstrated a linear decrease in the electrical conductivity of films after prolonged exposure to both humid air and H_2_O_2_/O_2_ (Figure 2b‐i).^[^ ^50^ ^]^ This loss of conductivity was attributed to the oxidation of MXene into a relatively less conductive anatase phase. The oxidation starts from the sheet edge, where vacant unterminated regions are fully exposed to oxidizing species (Figure 2b‐ii). Anatase formed on the edges initially retards the oxidation but does not fully passivate the material. The reaction is dampened only to a certain extent and eventually proceeds at a constant rate because the oxidizing species directly diffuse through the MXene/anatase interface.^[^ ^50^ ^]^

#### 2.1.2 Early Studies on Factors Influencing Ti_3_C_2_T_*x*_ Oxidation

The earliest extensive report on the oxidation stability of colloidal MXene was conducted by Zhang et al.^[^ ^33^ ^]^ Pristine MXene sheets with an average lateral size of 600 nm were left standing in the open air at RT for 1 week. “Branches” appeared at the edges and anatase nanoparticles (2–3 nm in size) formed at the basal planes of the sheets. They found that the edge sites are more prone to oxidation, and the branches grow from the edges to the basal planes of MXene sheets (Figure 2c‐i), which is consistent with the findings of Lipatov et al. (Figure 2b‐ii).^[^ ^50^ ^]^ They observed that the complete decomposition of MXene occurs after 30 days, resulting in a final material composed of anatase and disordered carbon (Figure 2c‐ii). In addition to oxidation studies, this work also developed a strategy to minimize the observed degradation of MXene. Here, they have demonstrated that the formation of anatase could be mitigated by storing the colloidal solutions in hermetic Ar‐filled bottles at 5 °C to remove dissolved oxygen, which was then considered the primary oxidant of MXene (Figure 2c‐iii).^[^ ^33^ ^]^

Building on this concept, Alhabeb et al.^[^ ^14^ ^]^ published a comprehensive guideline for the synthesis and processing of MXene in 2017. When aqueous MXene dispersions were left uncovered at room or elevated temperatures, anatase formation was observed within a week after synthesis, four times faster than the previous observations of Zhang et al. (30 days).^[^ ^14^ ^]^ In addition to limiting the interaction of MXene with dissolved O_2_ via Ar purging, another protocol was developed. This involved decreasing the storage temperature and eliminating UV exposure by refrigerating Ar‐purged MXene solutions, extending the shelf life to a minimum of 24 days.

A theoretical perspective on the oxidation mechanism of MXene was also demonstrated through MD simulations with ReaxFF as a forcefield. Analyzing the impact of various oxidizing agents, namely, dry air (O_2_), moist air (O_2_ and H_2_O), and hydrogen peroxide (H_2_O_2_), revealed that under similar conditions, the oxidation rate of MXene is highest in the presence of H_2_O_2_, followed by H_2_O and O_2_. Simulations of oxidation in humid conditions showed that some Ti atoms underneath the T layer are pulled by water toward the MXene surface, resulting in functionalization of the surface with –O and –OH groups.^[^ ^51^ ^]^

#### 2.1.3 Early Strategies to Prevent Degradation via Organic Solvent Redispersion

These early studies on dissolved O_2_ as the driving force of oxidation^[^ ^47^ ^]^ were later referred to by Maleski et al. in 2017. They developed organic solvent‐based systems for storing MXene, which also served as a method of preventing oxidation and extending its shelf life (Figure 2d).^[^ ^22^ ^]^ However, despite higher levels of dissolved oxygen being measured in ethanol and other organic solvents, anatase formation was still most prominent in water. This observation led to a hypothesis that MXene oxidation is not exclusively driven by dissolved O_2_ but also by H_2_O or H_2_O/O_2_.^[^ ^51^ ^]^ In fact, the earliest experimental observation of titanium carbide hydrolysis was made in 1967 by Avgustinik et al.^[^ ^52^ ^]^ and that the hydrolysis results in the formation of methane and hydrated TiO_2_. Contemporary studies have already observed slow water‐induced oxidation at the top atomic layers of bulk TiC,^[^ ^51^, ^53^ ^]^ but the literature has yet to produce direct studies to determine the influence of water toward MXene oxidation at that time.

### 2.2 (2019–2021) Influence of MXene Structural/Atomic Defects, Surface Chemistry, and Storage Conditions

#### 2.2.1 Influence of Structural/Atomic Defects

**Figure 3:**
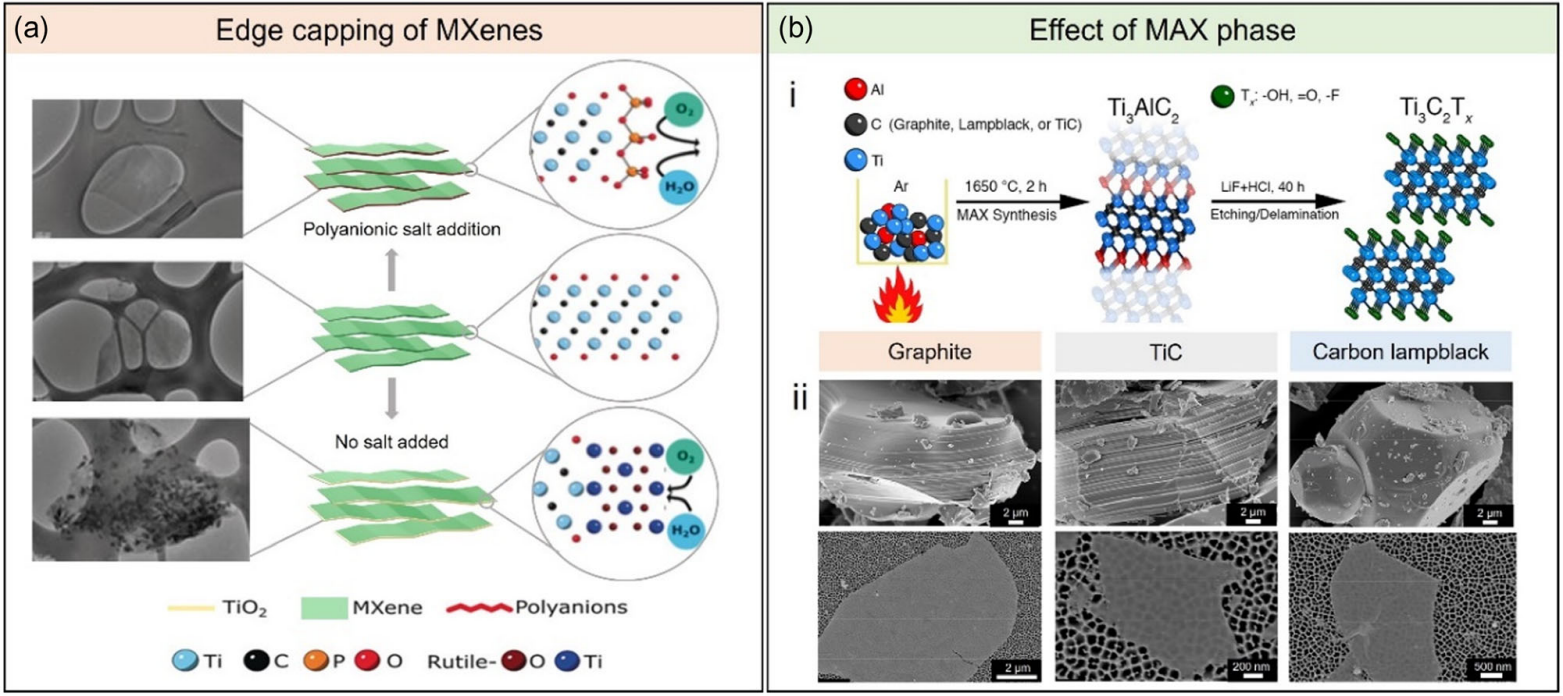
a) Simple edge capping strategy of individual MXene sheets in aerated water using polyanionic salts that prevent oxidation. Adapted with permission.^[^ ^57^ ^]^ Copyright 2019, Wiley‐VCH. b) A study on the effect of MAX phase precursors on MXene structure and properties. (i) Conditions of MAX phase synthesis using different carbon sources (TiC, graphite, and carbon lampblack). (ii) SEM images of the graphite‐, TiC‐, and carbon lampblack‐derived MAX phases synthesized using MILD‐like process. Adapted with permission.^[^ ^58^ ^]^ Copyright 2019, American Chemical Society.

While studies prior to 2019 have investigated the influence of dissolved oxygen and solvents, studies on how defects and surface chemistry contribute to the oxidation process are still yet to be explored. Xia et al. used aberration‐corrected atomic‐resolution scanning transmission electron microscopy (STEM) to probe the role of Ti vacancies and surface/edge defects as preferential sites for oxidation.^[^ ^37^ ^]^ When Ti atoms in MXene react with O_2_ to form anatase, electrons accumulate on the MXene surface at the (0001) plane, promoting the nucleation and growth of anatase on the (101) plane. The internal electric field caused by the separation of electrons and electron holes enhanced the overall Ti ion diffusion, hastening the oxidation of MXene. In addition to oxygen, they also acknowledged that certain solvents apart from water can facilitate oxidation. For instance, protic solvents like methanol, ethanol, and isopropanol are known to dissolve molecular oxygen even at RT, as well as stabilize highly unstable oxide and oxide‐derived radicals,^[^ ^54^ ^]^ which may be detrimental to the stability of MXenes. Furthermore, there is another report^[^ ^55^ ^]^ on improved O_2_ solubility in systems containing water and acetonitrile, a polar aprotic solvent. It was then proposed that mitigating oxidation can be achieved by 1) synthesizing high‐quality MXene with minimal structural defects; and 2) storing them under chemical and/or environmental conditions that limit the exposure to water and oxide‐producing compounds (e.g., O_2_, H_2_O_2_). In contrast, with the aid of positively and negatively charged gold nanoparticles (AuNPs), the work of Natu et al.^[^ ^56^ ^]^ in 2018 put forward a different perspective showing the existence of a charge difference between the flake surface (negative) and edge (positive) of Ti_3_C_2_T_*x*_ MXene. It was suggested that the positively charged edge is attributed to the exposed Ti atoms, which have less coordination than what is required to achieve electrical neutrality. Building from this insight, a follow‐up article^[^ ^57^ ^]^ proved that passivating the edge sites of MXene sheets with polyanionic salts suppresses MXene degradation (**Figure** **3**a). It was postulated that the edge of MXene flakes is most vulnerable to degradation due to the presence of the exposed Ti and C atoms, which are prone to attack by the dissolved O_2_. In a related study, Green et al. found out that the addition of antioxidants such as sodium L‐ascorbate (100 mg vs 0.6 mg MXene) prevented oxidation for up to 21 days.^[^ ^41^ ^]^ Aside from demonstrating how anionic edge capping passivates the MXene edge and mitigates oxidation, these studies also confirm that the positively charged MXene flake edges are highly susceptible to anion adsorption, which is inspiring for possible edge functionalization strategies.

Variations in the MAX phase precursors were also found to contribute to the oxidation resistance of the corresponding MXenes. In a study by Shuck et al. Ti_3_C_2_T_*x*_ MXene was etched from different MAX phases synthesized using three different carbon sources (graphite, carbon lampblack, and titanium carbide) at 1650 °C for 2 h.^[^ ^58^ ^]^ Thermal analysis revealed that the synthesized MAX phases underwent different reaction pathways, resulting in varying flake sizes, morphologies, electrical conductivity, and chemical stability in water of their corresponding MXenes (Figure 3b). Testing the resultant chemical stability against oxidation was carried out at low concentrations (0.15 mg mL^−1^) in water to accelerate degradation. The order of increasing stability of the aqueous MXene dispersions produced from different precursors is as follows: graphite (10.1 days) < TiC (time constant = 4.8 days) < carbon lampblack (5.1 days). A similar trend was also observed when comparing size and conductivity. This study serves as the first demonstration on how the carbon precursors of MAX phases affect the size, quality, and stability of the resulting MXene, identifying another critical variable that can be controlled and tailored for specific applications. In addition, this study also pointed out that the variations in MAX phase preparation across different laboratories, (e.g., carbon precursor or other synthesis conditions) could significantly affect the quality of MXene even when using similar etching and processing methods.

#### 2.2.2 Early Tests Probing the Influence of Water on MXene Oxidation

**Figure 4:**
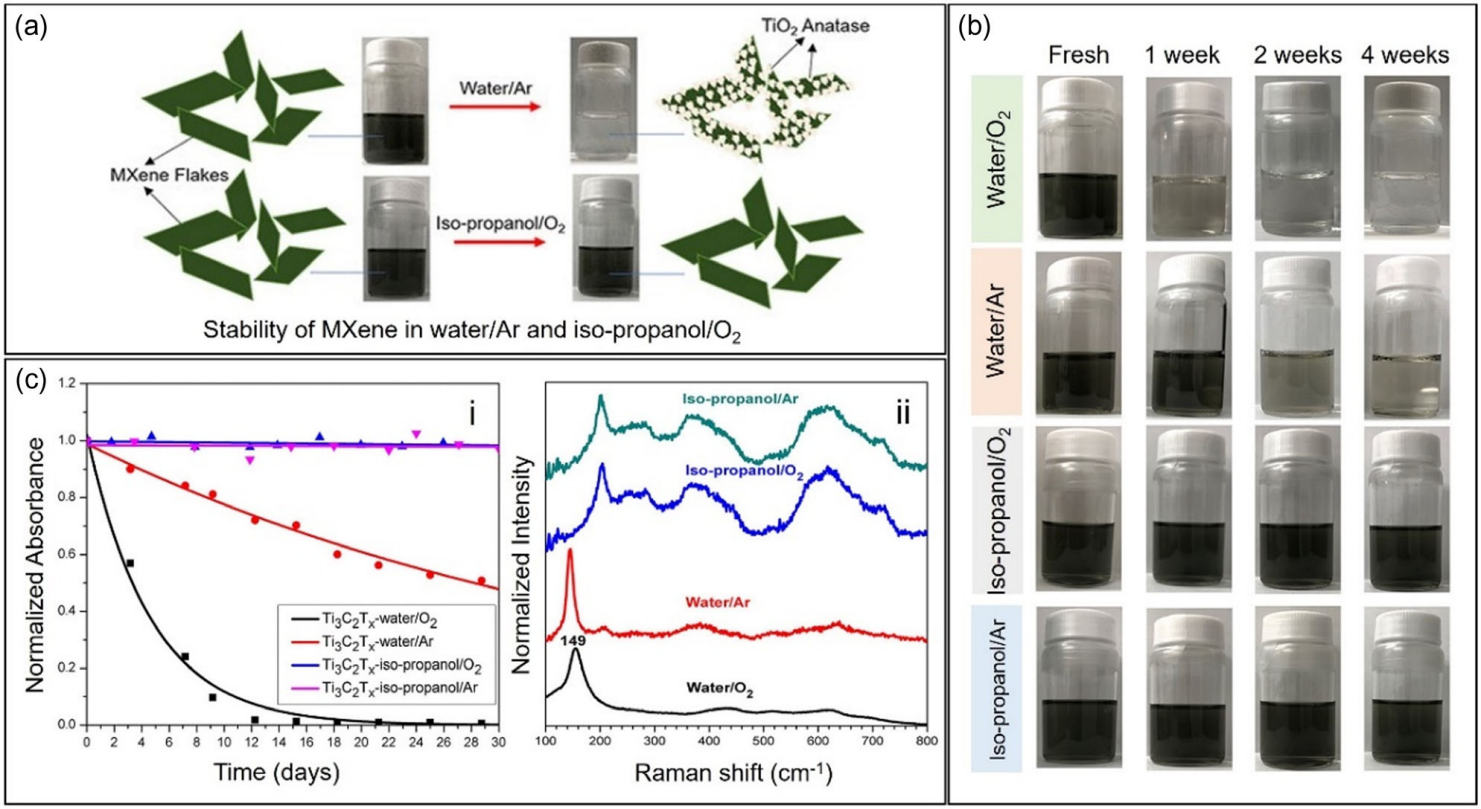
a) Diagram showing the oxidation of MXene dispersed in water/Ar where the formation of TiO_2_ was observed and the stability of MXene dispersed in isopropanol/O_2_. b) Colloidal solutions of MXene in different conditions over time. c) Continuous decrease in the MXene concentration over time as seen in the (i) degradation kinetics plot and (ii) Raman spectra. Adapted with permission.^[^ ^44^ ^]^ Copyright 2019, American Chemical Society.

Direct experiments on determining if water is the primary driver of MXene oxidation were carried out in 2019 by Huang et al. (**Figure** **4**a).^[^ ^44^ ^]^ Although water‐induced oxidation has been observed since 2014,^[^ ^22^, ^47^, ^50^ ^]^ this is the first study to demonstrate that oxidation proceeds even in the absence of dissolved oxygen. MXene dispersions were subjected to different conditions, including exposure to O_2_ or Ar and/or dispersion in water or isopropanol (Figure 4b). The degradation of MXene in these samples was verified by observing a continuous decrease in MXene concentration over time, as seen in the degradation kinetics plot (Figure 4c‐i). Furthermore, characteristic Raman signals in the range of 149–155 cm^−1^ for both aqueous dispersions denote the abundant presence of TiO_2_, while the MXene signals have disappeared, which is the opposite case for the isopropanol dispersions (Figure 4c‐ii). Interestingly, the oxidation process was slower when MXene is dispersed in O_2_‐purged isopropanol  than in Ar‐purged water. This indicates that dissolved oxygen has a significantly lower contribution than water to the overall degradation process at ambient conditions. This was the first study that independently illustrated the effects of both water and atmospheric O_2_ in the degradation of MXene, pointing at hydrolysis as an alternative and, based on their findings, a much faster degradation route. Furthermore, it was suggested that preventing contact between MXene sheets and water is a more significant mitigation strategy than minimizing interaction with O_2_.

#### 2.2.3 Freezing as a Preventive Measure to Minimize Contact with Water

In the same year, Chae et al. reported that freezing water can improve the oxidation stability of MXene. Storing aqueous MXene dispersions at −80 °C can mitigate the degradation process for more than 39 weeks.^[^ ^59^ ^]^ Furthermore, they found that dispersing MXene in a nonaqueous medium (ethanol) also delayed the degradation, even when stored at a higher temperature of 5 °C. This further supports the hypothesis that degradation can be mitigated when water is absent.

**Figure 5:**
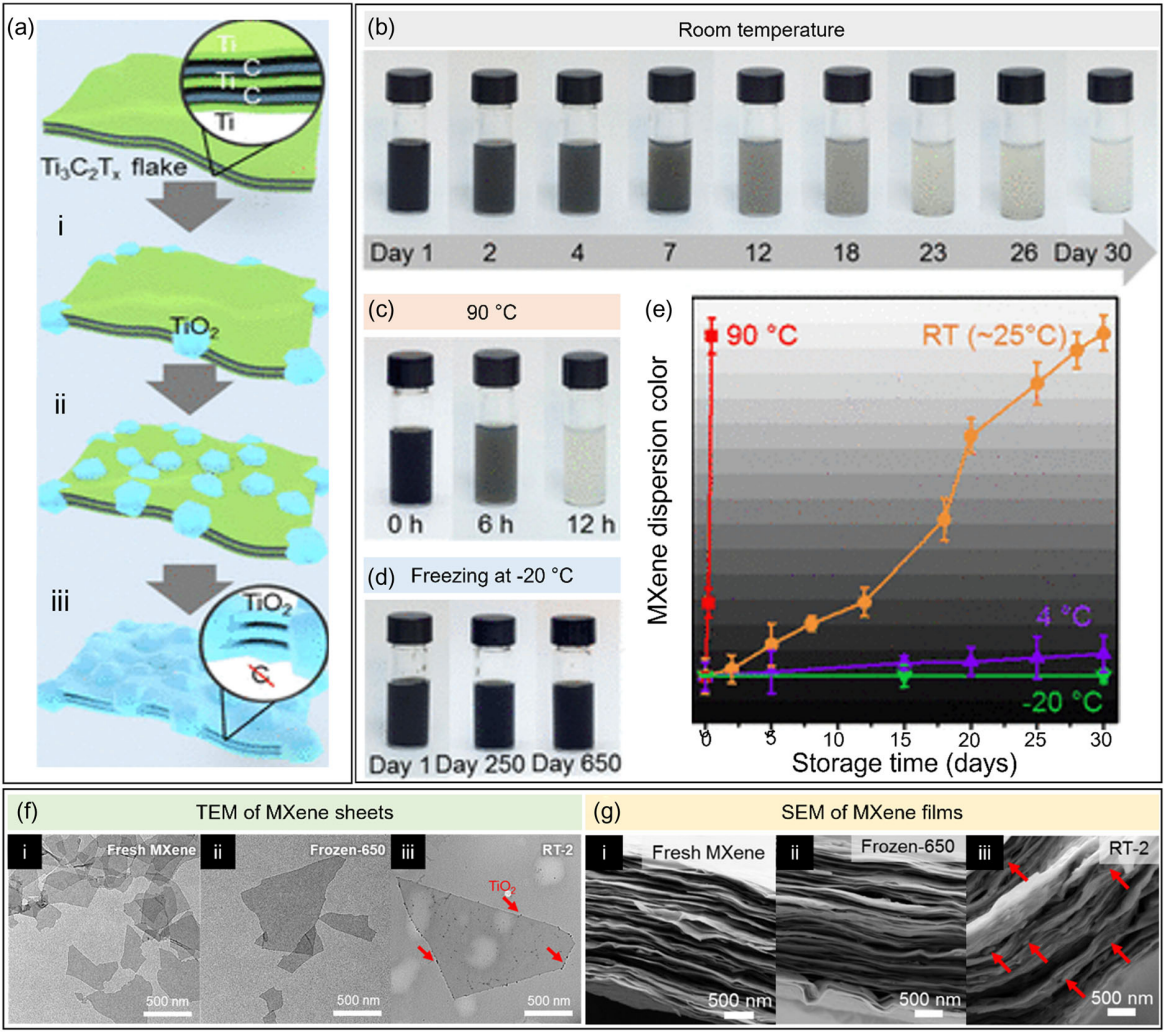
a) Schematic illustration of the oxidation process of single MXene sheets in dispersion. b) Photos of the aqueous MXene dispersion (0.5 mg mL^−1^) stored at RT(≈25 °C) over 30 days; c) incubated in an oven at 90 °C for 0, 6, and 12 h; and d) stored at −20 °C for 250 and 650 days. e) Color fading of MXene dispersions stored at different temperatures as a function of storing time, in which the data point was obtained by digital analysis of the photograph of MXene dispersions using ImageJ. The background is a dark green step wedge linearly changed from *R*/*G*/*B* = 4:4:4 (bottom) to *R*/*G*/*B* = 247:247:247 (top), which works as the *y‐*axis. The error bar is derived from the variation of colors at different positions of the dispersion in the photographs. f) TEM nanosheet images and g) SEM film images of (i) fresh MXene, (ii) MXene frozen at −20 °C for 650 days, and (iii) MXene stored at RT for 2 days. Adapted with permission.^[^ ^40^ ^]^ Copyright 2020, American Chemical Society.

The conversion of MXene to anatase is known to be a temperature‐dependent process, with oxidation inevitable at RT (**Figure** **5**b) and increases at elevated temperatures (Figure 5c). From this knowledge, Zhang et al.^[^ ^40^ ^]^ demonstrated how storing MXene dispersions at –20 °C extended the storage life of MXene up to 650 days (Figure 5d). A visual indicator is the minimal color change (dark to gray) of the frozen dispersions compared to those stored at RT and at 90 °C (Figure 5e). Several imaging techniques also visualized the progression of this oxidation process. First, transmission electron microscopy (TEM) (Figure 5f) and atomic force microscope (AFM) images revealed a noticeable TiO_2_ formation at the edges of individual MXene flakes acquired from aqueous dispersions stored at RT for 2 days (denoted as RT‐2). Meanwhile, a clean surface and edge morphology was observed for both fresh MXenes, as well as those frozen for 650 days (denoted as Frozen‐650). Furthermore, cross‐sectional scanning electron microscopy (SEM) images of the films (Figure 5g) also reveal particle formation between the overlapping flakes of RT‐2 films, while no small particles were observed for both the fresh and Frozen‐650 MXenes. These findings prove that a general laboratory freezer suffices for long‐term MXene storage in the aqueous medium.

#### 2.2.4 Environmental Factors Affecting MXene Oxidation Kinetics

**Figure 6:**
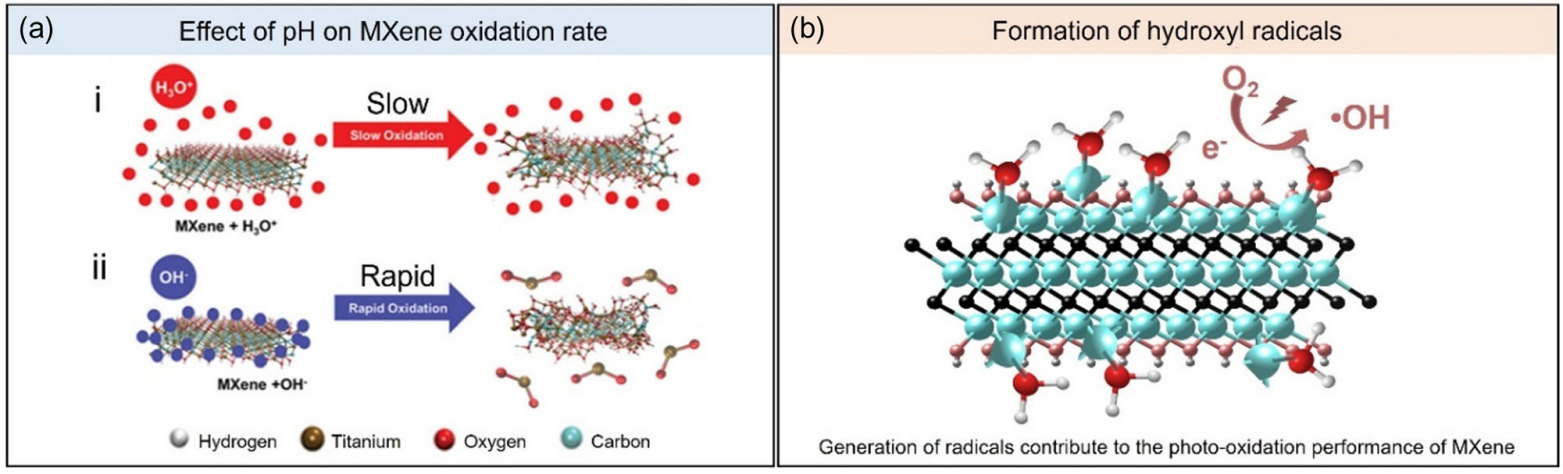
a) Schematic diagram of the (i) slow and (ii) rapid oxidation of aqueous MXene dispersions in acidic and alkaline pH, respectively. Adapted with permission.^[^ ^60^ ^]^ Copyright 2020, Wiley‐VCH. b) Generation of hydroxyl radicals by MXene nanosheets under UV light that contributes to the oxidation process.^[^ ^61^ ^]^

In a study conducted by Zhao et al. in 2020, the oxidation kinetics were found to be dependent on the pH, temperature, and concentration of the MXene dispersion.^[^ ^60^ ^]^ MXene nanosheets interact rapidly with hydroxide ions in basic medium, forming deprotonated oxide (–O^−^) terminal groups and making MXene more prone to oxidation. Moreover, the higher the availability of hydroxyls, the faster the oxidation rate becomes. To counteract this phenomenon, they proposed using citric acid as an antioxidant to mitigate MXene oxidation. They also observed that higher MXene concentrations slow down oxidation compared to dispersions stored in low concentrations. Based on the findings, they suggested that when the MXene concentration is high, each sheet cause a steric shielding effect due to the close proximity of the flakes, limiting the access of water to the surface and edges of MXene flakes, thus reducing the degradation rate. Furthermore, they proposed how the critical concentration (c*), the point at which the flakes begin to exhibit this effect, can be calculated based on the average size and thickness of the flakes, which can be measured using AFM. In summary, they suggested that MXene dispersions can be best stored in high concentration, acidic pH, and with citric acid (**Figure** **6**a).

In a study by Rosales et al. it was reported that MXene has the ability to generate hydroxyl radicals (•OH) from its defective surface Ti sites under UV light (Figure 6b).^[^ ^61^ ^]^ Single‐layered MXene nanosheets have more available active anatase sites. These sites can generate four times more •OH than the multilayered MXenes under UV light. Although there are still no direct studies on the influence of these radicals on MXene oxidation, it has been reported that the presence of such species contributes to other oxidation processes.^[^ ^61^, ^62^, ^63^ ^]^

#### 2.2.5 Systematic Identification of Oxidation By‐Products

**Figure 7:**
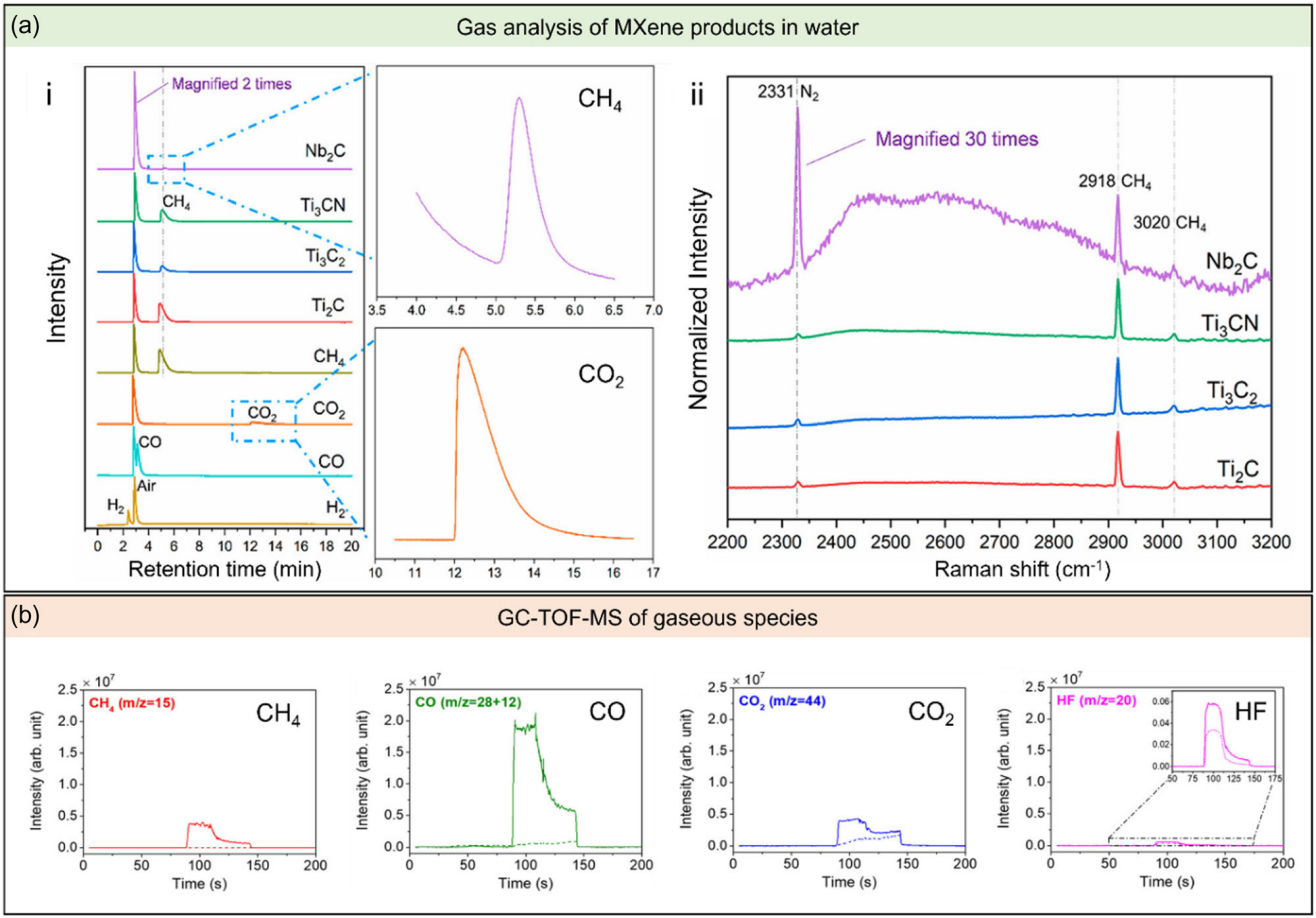
a) Analysis of the gaseous reaction products of MXene in aqueous media. (a) GC results of the different gas products with the standard gases (H_2_, CO, CO_2_, and CH_4_). Images on the right show the peak areas of CH_4_ produced after degradation and the CO_2_ peak in reference to the standard CO_2_ gas. (ii) Raman spectra of the gas bubbles produced after MXene transformation in aqueous solutions. Adapted with permission.^[^ ^36^ ^]^ Copyright 2020, American Chemical Society. b) GC‐TOF‐MS chromatograms of the gases (CH_4_, CO, CO_2_, and HF) produced during oxidation at pH 5.4 and 20 °C. For reference, the dashed and solid lines represent the intensity of gases before and after oxidation, respectively. Adapted with permission.^[^ ^38^ ^]^ Copyright 2021, American Chemical Society.

Previously, it was known that the oxidation of MXene yields defective flakes and nanosized anatase TiO_2_ and amorphous carbon as the main by‐products.^[^ ^33^, ^50^ ^]^ However, Huang et al. demonstrated using Raman spectroscopy and gas chromatography (GC) that some gases are also produced during MXene degradation.^[^ ^36^ ^]^ A glass headspace vial containing aqueous MXene dispersion was placed inside an oven at 70 °C to accelerate the process.^[^ ^33^, ^36^, ^50^ ^]^ Both the GC and Raman spectroscopy data observed methane (CH_4_) as one of the degradation products of MXene in water (**Figure** **7**a‐i to ii). Even though CO and CO_2_ were predicted to be present in the oxidative degradation of MXenes, these gases were barely detected (Figure 7a‐i). In addition, Raman and energy‐dispersive X‐ray spectroscopy analysis of the solid oxidation products confirmed the formation of anatase, but no trace of carbon was detected. The authors concluded that MXene in aqueous media mainly transforms into anatase and CH_4_.

Doo et al. also studied the products of MXene oxidation at varying pH and temperature.^[^ ^38^ ^]^ At pH 5.4 and 20 °C, the degradation products include anatase and amorphous carbon. Additionally, other gaseous species, such as CH_4_, CO, CO_2_, and HF were detected using gas chromatography ‐ time‐of‐flight ‐ mass spectrometry (GC‐TOF‐MS) (Figure 7b). The degree of oxidation was monitored through the visual changes in the suspension, which evolved from dark to cloudy, providing evidence of anatase formation. This was further confirmed using UV–vis spectroscopy (*λ*_max_ at 780 nm). As the reaction was allowed to proceed, the pH of the MXene dispersion slowly decreased. This drop in pH is caused by an increase in H^+^ concentration resulting from the presence of dissolved CO_2_ and HF by‐products in the aqueous solution.

#### 2.2.6 Degradation under Acidic and Alkaline Conditions

**Figure 8:**
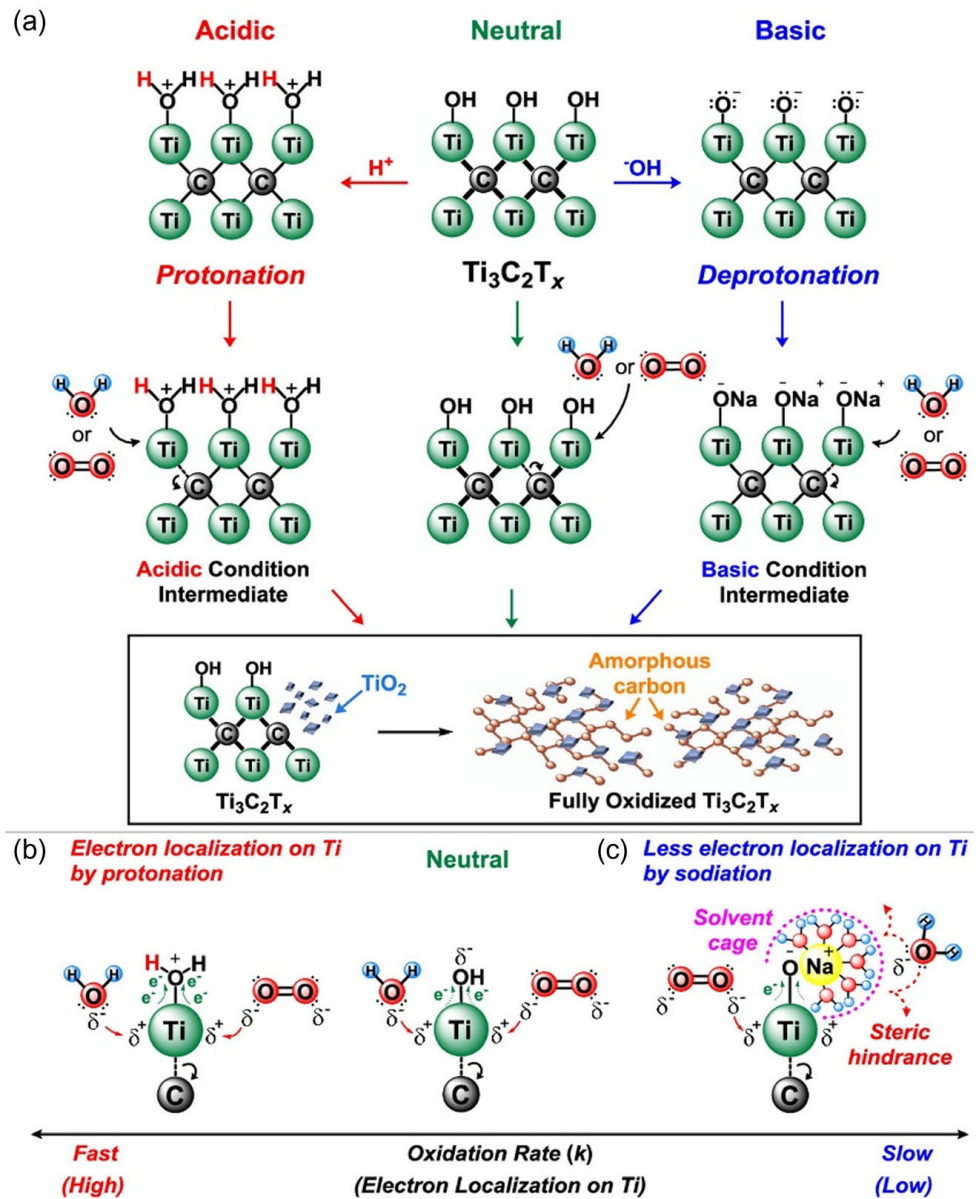
a) Proposed mechanism of the oxidation reaction of MXene dispersions under b) acidic and c) basic conditions. Adapted with permission.^[^ ^38^ ^]^ Copyright 2021, American Chemical Society.

In the study of Doo et al. it was observed that the reaction rate gradually slows when the pH is increased at a constant temperature of 20 °C,^[^ ^38^ ^]^ suggesting that basic conditions decelerate MXene oxidation. These findings contradict the observations of Zhao et al. in 2020.^[^ ^60^ ^]^ This combination of experimental and computational studies proposed a degradation mechanism via an acid‐catalyzed reaction, where the hydroxyl groups on the surface of MXene are protonated by H^+^ ions (**Figure** **8**a,b). The electrons in the Ti—O bonds are drawn toward O rather than Ti, increasing the susceptibility of the electron‐deficient Ti intermediate to attack by water or oxygen. Under basic conditions, the OH^−^ ions deprotonate the hydroxyl group on the surface of MXene, resulting in an enolate (Ti—O—) moiety (Figure 8a,c) that can be stabilized by Na^+^ ions. The steric effect from the size of the Na^+^ ion then prevents the attack of water or oxygen. Both studies have provided plausible oxidation mechanisms at various pH conditions. However, the conflicting results presented by the abovementioned studies call for a more in‐depth examination beyond understanding just the effect of surface terminations, which may involve probing changes within the MXene structure.

#### 2.2.7 Preventing Water Contact by Utilizing Salt Hydration Chemistry

**Figure 9:**
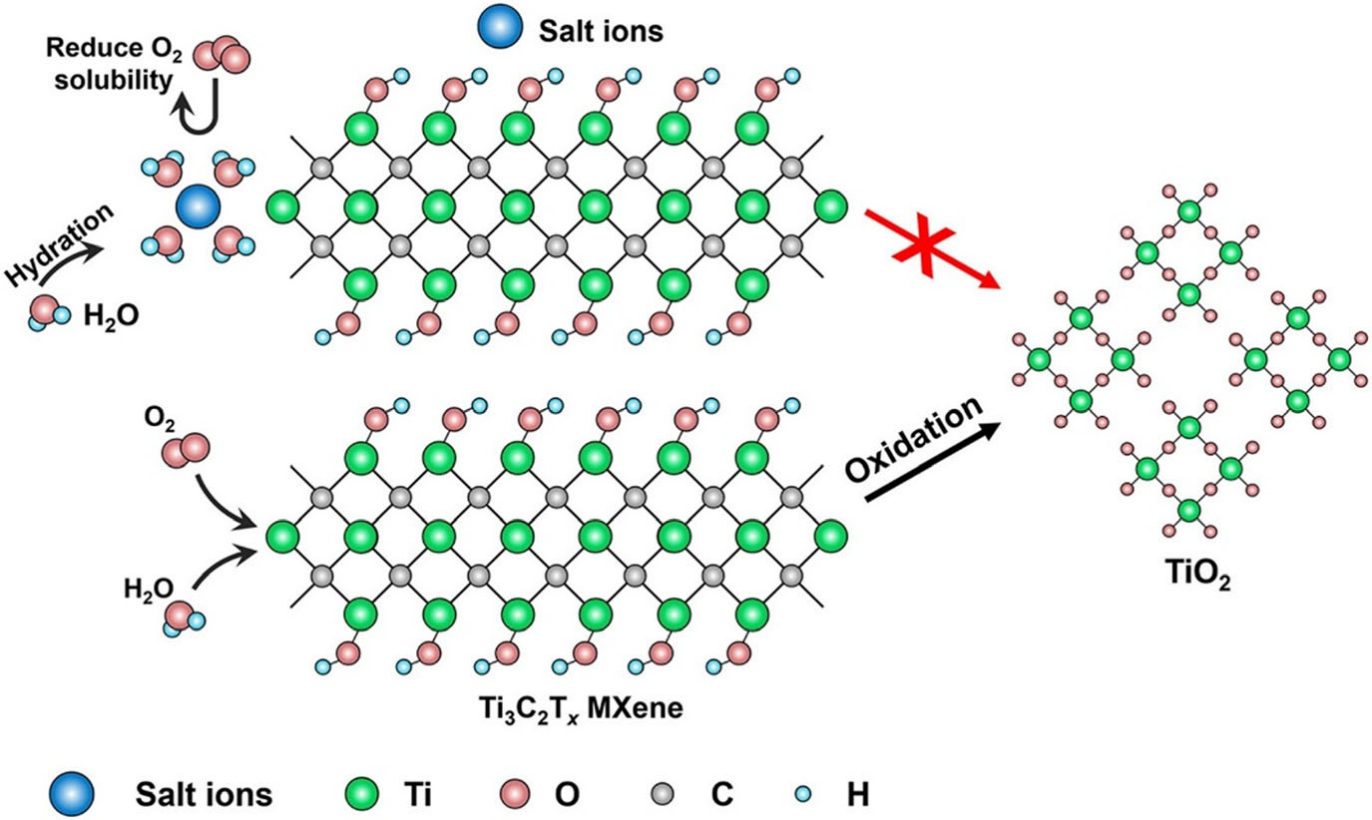
Schematic illustration of the protection of MXenes by hydration of salt ions. Adapted with permission.^[^ ^64^ ^]^ Copyright 2021, Wiley‐VCH.

By 2021, a method for the long‐term storage of MXene in aqueous solution based on the hydration chemistry of nontoxic inorganic salts (NaCl, LiCl, and CaCl_2_) was developed.^[^ ^64^ ^]^ The interaction of MXene with free water and dissolved oxygen was inhibited by the hydration of the salts (**Figure** **9**). This study reported that aqueous MXene dispersions can be stored for up to 400 days in saturated saline solution without sacrificing their intrinsic properties. The authors claim that salt protectants can be easily removed upon use. The minimal amount of free water in the solution limits the presence of dissolved oxygen that contributes to the suppression of MXene oxidation.

### 2.3 (2022–Present) Water as Main Driver of Degradation—Efforts to Mitigate Hydrolysis and Advancements in Probing Oxidation Propagation in MXenes

#### 2.3.1 Simulations Supporting Hydrolysis as the Driver of MXene Degradation

**Figure 10:**
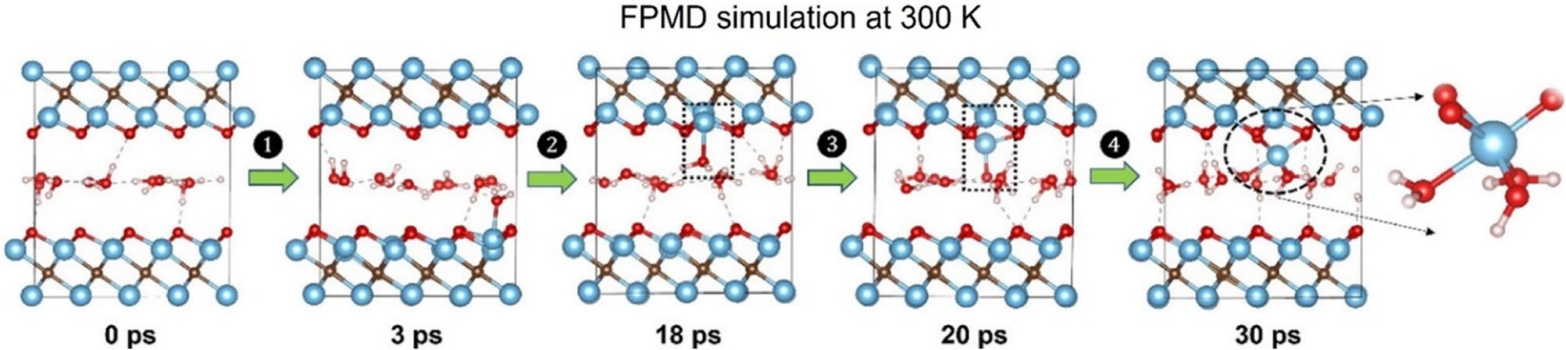
Representative snapshots for one layer of water confined in Ti_3_C_2_O_2_ during the 30 ps FPMD simulation at 300 K, demonstrating the four stages of the process when water attacks the surface of Ti_3_C_2_O_2_: (1) reversible water adsorption on Ti, the stage where water comes on and off a surface Ti atoms; (2) irreversible water adsorption, the stage where water sticks on the surface and does not come off the Ti atom; (3) water dissociation and Ti pullout; and (4) further reaction of water molecules with the pullout Ti. Color code: Ti, blue; O, red; C, gray; and H, white. The same color scheme was used subsequently. Adapted with permission.^[^ ^53^ ^]^ Copyright 2022, American Chemical Society.

The presence of water‐promoting oxidation in MXene dispersions (in addition to or in parallel with oxygen) has been noted in the literature even prior to 2017,^[^ ^22^, ^44^ ^]^ but the initiative toward understanding whether water or oxygen mainly drives this reaction did not start until the systematic study of Huang et al. was published in 2019.^[^ ^44^ ^]^ However, the mechanism of water‐induced degradation of MXenes remained unclear. Wu et al. first simulated this process in 2022 through first‐principle molecular dynamics (FPMD) simulations at RT using a system of water and Ti_3_C_2_O_2_ MXene sheets.^[^ ^53^ ^]^ In this study, it was shown that water molecules attack the basal plane of Ti_3_C_2_O_2_ via irreversible adsorption on the surface Ti atoms, followed by deprotonation and Ti pullout by the adsorbed —OH group occurring simultaneously with Ti—C bond breaking (**Figure** **10**). The FPMD results supported the experimental observations of MXene hydrolytic degradation in previous studies, including anatase formation and acid‐catalyzed degradation. However, they did not clarify why this oxide formation is more visible on the sheet edge. Nevertheless, FPMD simulations still have limitations, such as system size and the accessible timescale, which further studies can account for. A longer timescale is recommended for studying continuous water attacks until the presumed eventual termination of this process. Additionally, the simulation used Ti_3_C_2_O_2_, which has a more ordered surface compared to the mixed terminations in Ti_3_C_2_T_*x*_, and may not fully represent the surface properties of MXenes typically produced in the laboratory.

#### 2.3.2 New Mitigation Strategies to Minimize Water‐Induced Oxidation

**Figure 11:**
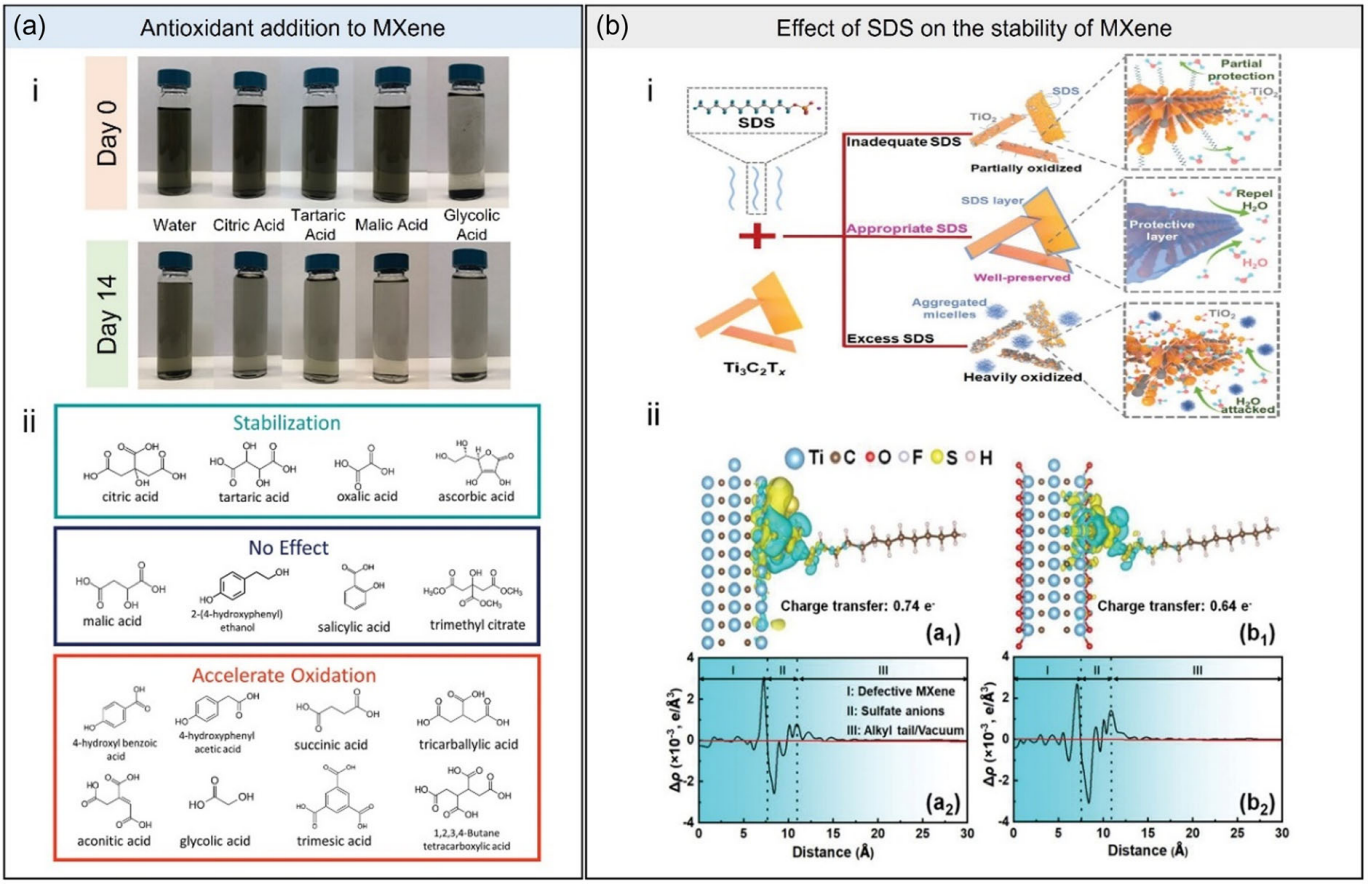
a) Antioxidant addition to MXene. (i) Digital images of MXene dispersions before and after storage. (ii) Summary of effects of antioxidant candidates on MXene dispersion stability.^[^ ^39^ ^]^ Copyright 2022, Wiley‐VCH. b) Effect of SDS on the stability of MXene. (i) Schematic illustration of SDS‐induced antioxidation capability in MXene dispersion at varied SDS concentrations. (ii) Charge density difference analyses (subscript 1) on the adsorption of SDS on a) group‐free Ti_3_C_2_ and b) defective Ti_3_C_2_O_2_ surfaces (isosurface: 0.005 e Bohr^−3^) accompanied by the corresponding planar‐average charge density difference (Δ*ρ*, subscript 2) along the z‐direction. Adapted with permission.^[^ ^65^ ^]^ Copyright 2023, Wiley‐VCH.

Since 2022, most mitigation strategies for MXene oxidation have shifted their focus on minimizing hydrolytic attacks on the sheets. Antioxidants, such as sodium L‐ascorbate, ascorbic acid, and polyanions, are still effective mitigation strategies (**Figure** **11**a‐i).^[^ ^39^ ^]^ In contrast to early studies, recent works have highlighted how antioxidants chelate with the Ti atoms in MXene, preventing their interaction with water. Researchers examined antioxidants with varying degrees of mitigation against MXene oxidation, which are thus classified as 1) stabilizing, 2) no effect; and 3) accelerated oxidation (Figure 11a‐ii). It is interesting to note that while some antioxidants provide no protection, others even accelerate MXene oxidation. The nature of the interaction between these antioxidants and MXene, whether permanent or temporary, is still unclear and needs further research.

Another capping agent explored by Fan et al. in 2023 was sodium dodecyl sulfate (SDS).^[^ ^65^ ^]^ At a concentration of 1.5 mg mL^−1^, SDS delayed the Ti_3_C_2_T_*x*_ MXene oxidation for up to 213 days, suggesting that it is possibly due to the SDS molecules adsorbing onto both the surface and edges of MXene, preventing direct water contact on the sheet surface and the defective sites. The researchers also investigated the effects of “underprotecting” and “overprotecting” the MXenes with the surfactant (Figure 11b‐i). MXenes that were underprotected, dispersed in less than 1.5 mg mL^−1^ of SDS, were partially oxidized. Conversely, an excess of SDS formed micelles filled with water instead of being adsorbed on the surface of MXene, thereby promoting oxidation. It was predicted that the negative stabilizing effect of SDS at 2 mg mL^−1^ was brought by its poor dispersibility beyond the critical micelle concentration (1.91 mg mL^−1^ in water), which reduces the amount of SDS molecules interacting with MXene. Monte Carlo and MD simulations, as well as negative energy values (*E*_inter_) acquired from DFT calculations, revealed a spontaneous SDS‐MXene interaction, especially on highly positive defect sites. The migration of electrons from the sulfate terminal of SDS to the positively charged defects provides a reducing environment that passivates the surface and edges from oxidizing species (Figure 11b‐ii).

#### 2.3.3 Understanding the Propagation of Oxidation in the MXene Crystal Structure

**Figure 12:**
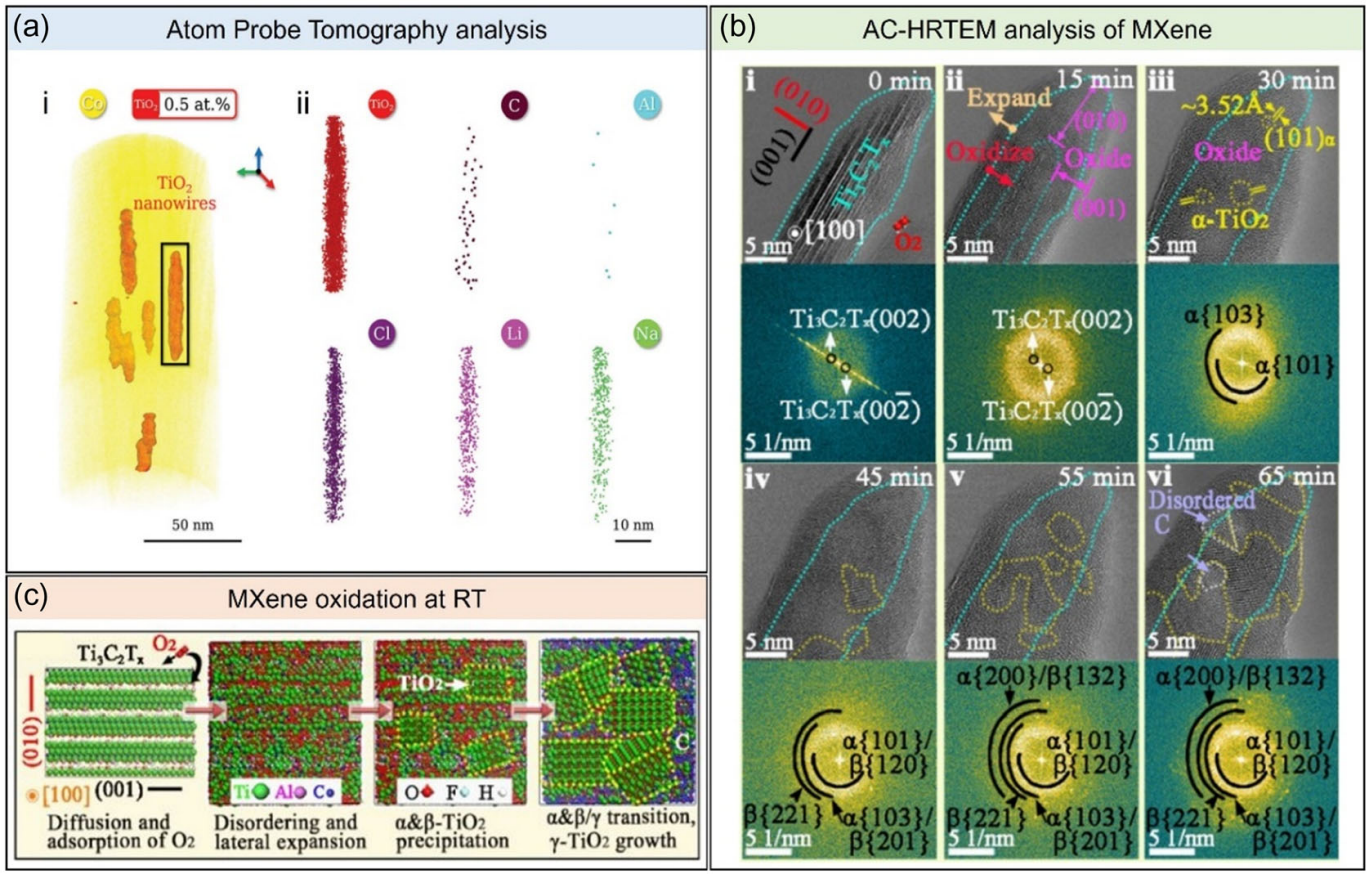
a) APT analysis of oxidized MXenes. (i) Reconstructed 3D atom map. TiO_2_ nanowires are highlighted by red isocompositional surfaces at 0.5%. (ii) Molecular distribution map of TiO_2_ and elemental distribution maps of impurity elements in the extracted region of interest indicated in (i). Adapted with permission.^[^ ^66^ ^]^ Copyright 2024, Wiley‐VCH. b) Time‐resolved cross‐sectional aberration‐corrected high‐resolution transmission electron microscope (AC‐HRTEM) images of MXene oxidized for (i) 0 min, (ii) 15 min, (iii) 30 min, (iv) 45 min, (v) 55 min, and (vi) 65 min. Indexed fast Fourier transform is shown below each HRTEM image. The cyan dotted lines indicate the original position of the MXene surface before oxidation. The semitransparent cyan dotted line in (ii) indicates the interface between MXene and oxide. Adapted with permission.^[^ ^67^ ^]^ Copyright 2024, Wiley‐VCH. c) Schematic illustrations of the evolution of MXene upon oxidation at RT. AO, area of oxide. Adapted with permission.^[^ ^67^ ^]^ Copyright 2024, Wiley‐VCH.

Several strategies have been developed to more efficiently cap MXene, preventing contact with water and thereby avoiding hydrolysis and oxidation. However, the mechanism of anatase formation and its progression from the edge to the basal plane remained underexplored. In a study by Kramer et al. in 2023, atom probe tomography (APT) was utilized to reveal the presence of alkali metal (Li, Na) and halogen (Cl, F) surface terminations, intercalated ions, and impurities introduced during the wet chemical synthesis of MXene.^[^ ^66^ ^]^ The analysis demonstrated how the presence of these alkali elements could serve as nucleation sites for anatase growth during the oxidation process (**Figure** **12**a). These positively charged alkali metals stabilize the oxide and attract more water molecules to the MXene sheets. However, the dependence of APT resolution on factors such as sample preparation, in addition to being a relatively unutilized technique for MXenes, resulted in challenges in providing information on whether the alkali impurities were adsorbed on the surface or integrated into the single‐layer structure of MXene.

A more recent study by Liu et al. employed several experimental and theoretical techniques to elucidate the early stages of oxidation in HF‐etched MXene under O_2_ at RT.^[^ ^67^ ^]^ Using aberration‐corrected environmental TEM and reactive MD simulations, they determined that the oxidation rate of MXene is highly dependent on the exposed crystal plane. Specifically, the (010) and (100) planes of MXene exhibit higher oxidation rates than the (001) plane (Figure 12b). Moreover, an apparent expansion of the (001) plane of MXene during oxidation was attributed to the diffusion of Ti atoms into the subsurface layer beneath the surface O ridge, then toward the surface to react with O. The anatase and brookite TiO_2_ phases then successively precipitated from the amorphous region of oxidized MXene, growing irregularly and transforming into rutile. However, it must be noted that the investigation was only limited to the sole influence of O_2_ on the rate of MXene oxidation, hence, necessitating the need for a similar study using water‐induced degradation pathways (Figure 12c). Meanwhile, another theoretical study by Nesterova et al.^[^ ^68^ ^]^ also suggested that the initial rate of oxidative degradation depends mainly on the initial surface composition and the amount of available coordination sites present in the surface Ti atom. Their analysis of various singly terminated Ti_3_C_2_T_*x*_ MXenes (T = O, OH, Cl, F) and those with purely hydroxyl and oxide (OH:O) terminations varied at different ratios (1:1, 2:1, 1:2) revealed that the T composition has a direct consequence to both the ease of formation of Ti vacancies and ease of H_2_O attack, although it was also noted that the complexity of the terminations could also complicate the analysis. Hence, further theoretical research built upon this groundwork is encouraged to establish a predictive model to account for the complex nature of MXene's surface terminations.

### 2.4 Follow‐Up Studies to Further Improve Current Mitigation Strategies

This section provides additional research that has contributed to a deeper understanding of the predominant MXene oxidation mechanisms and the refinement of existing mitigation strategies. The studies referenced here were published in subsequent years following the foundational works outlined in Section 2. They are included separately to avoid any confusion regarding the chronological order of discoveries in literature.

#### 2.4.1 Additional Studies on Solvent Protection of MXenes

It was established in Section 2.1.3 that redispersing MXene into organic solvents prevents oxidation for up to 28 days.^[^ ^14^, ^22^ ^]^ However, this method was developed during a period when MXenes were primarily synthesized as multilayer structures (via the HF method), and delamination methods were not yet optimized (<2017). Consequently, this method involved redispersing dried MXene powder in organic solvents with the aid of ultrasonication. Multilayer MXenes synthesized after 2017 were easier to delaminate into single‐layer sheets, but the resulting powders were denser and more challenging to redisperse.^[^ ^28^, ^43^ ^]^ This necessitated harsher sonication conditions, leading to decreased sheet size and increased damage and defects.

**Figure 13:**
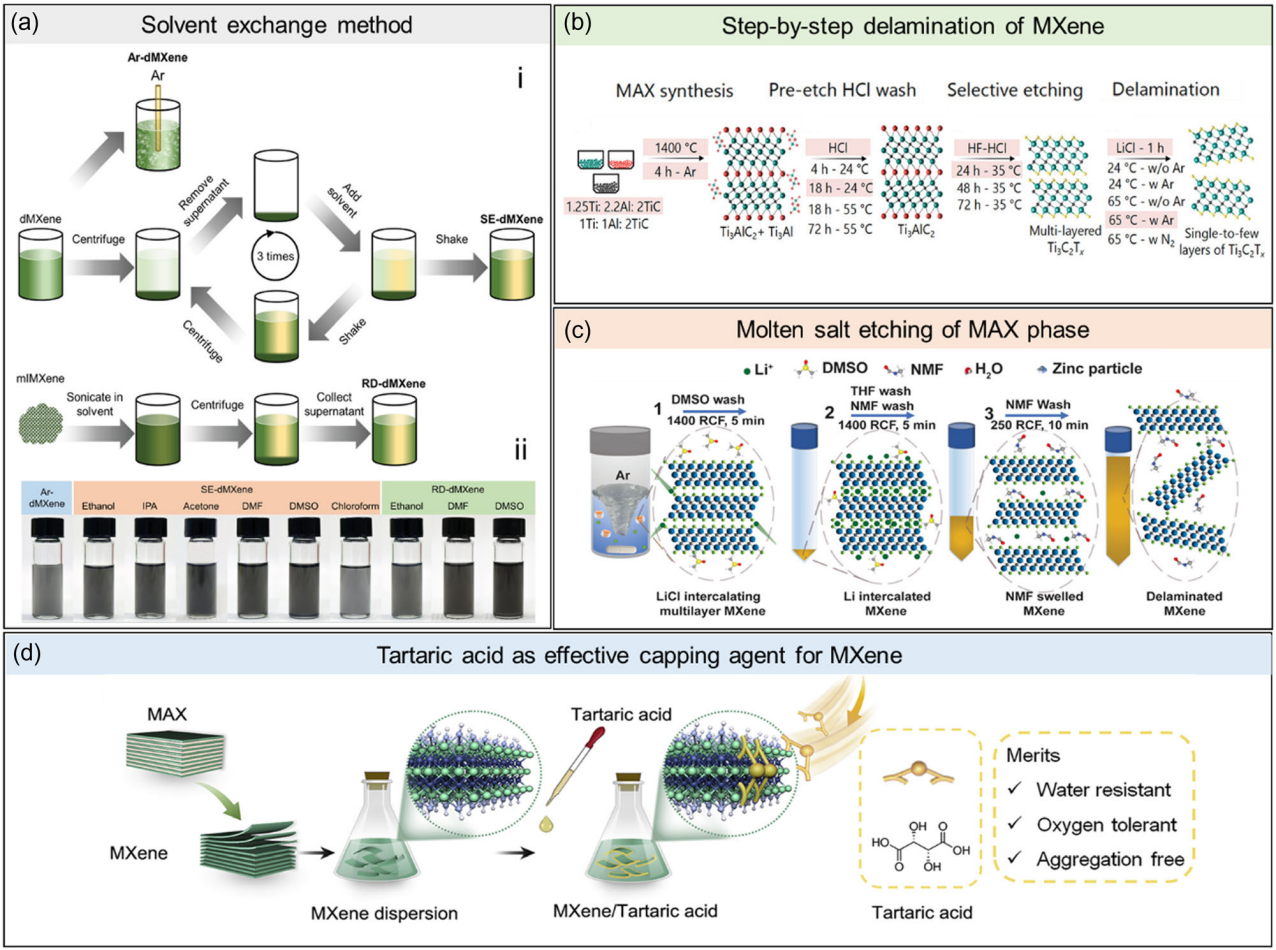
a) A schematic illustration of the SE process involving subsequent centrifugation cycles to remove the original solvent followed by replacement and MXene redispersion into the target system. Adapted with permission.^[^ ^43^ ^]^ Copyright 2019, Wiley‐VCH. b) A method for the delamination of MXenes derived from molten salt synthesis. Adapted with permission.^[^ ^72^ ^]^ Copyright 2024, American Chemical Society. c) A step‐by‐step protocol on synthesizing and etching of MXene from excess‐Al MAX phases. Adapted with permission.^[^ ^16^ ^]^ Copyright 2023, Wiley‐VCH. d) Schematic illustration of tartaric acid as capping agent for MXenes. Adapted with permission.^[^ ^74^ ^]^ Copyright 2024, Elsevier B.V.

One approach to overcoming these challenges was through the adaptation of the solvent exchange (SE) process. This method involved subsequent centrifugation cycles to remove the original solvent, followed by replacement and redispersion into the target solvent (**Figure** **13**a).^[^ ^43^ ^]^ Unlike previous MXene powders, the sediment acquired after centrifugation remained redispersible using the target organic solvent (e.g., DMSO, DMF, and NMP). The stability of these MXene dispersions was observed after storage at RT for up to 30 days and solution processibility was retained, as demonstrated in the fabrication of conductive fibers.^[^ ^43^ ^]^

#### 2.4.2 Modified MAX Phase Synthesis for Less Defective MXenes

Section 2.2.1 delineates how researchers uncovered the influence of defects and surfaces in MXene on the oxidation process. Since then, several methods have been developed to improve the quality of MAX phases for producing less defective MXenes. One method involves adding excess aluminum during the synthesis of the Ti_3_AlC_2_ MAX phase precursor, resulting in high‐quality MXene with conductivity measured at >20 000 S cm^−1^ and improved shelf life and oxidation stability even under ambient conditions.^[^ ^12^ ^]^ After storage in a closed vial at RT for 10 months, the synthesized MXene showed minimal degradation.

Although the presence of oxygen in bulk transition metal carbides and MAX phases has long been known, confirming and quantifying oxygen located within the layers of MXene has remained challenging due to the presence of oxygen in the surface termination layers and vacancies in the transition metal layers. However, a study by Michalowski et al. in 2022 confirmed that MAX phases produced using the typical stoichiometric ratio contained oxycarbides (Ti–C–O).^[^ ^69^ ^]^ The presence of oxygen in the Ti–C lattice provides nucleation points for anatase formation, rendering the resulting MXenes inherently prone to oxidation. This recent confirmation prompted the use of higher‐quality excess‐Al MAX phases containing minimal to no oxygen in the lattice, synthesized by Mathis et al. in 2021.

#### 2.4.3 Modified MXene Synthesis Methods Toward More Oxidation‐Resistant Surface Terminations

Aside from the quality of the MAX phase, etching conditions also play a key role in minimizing defects in MXenes. Further optimizations in the MXene etching method have been investigated to obtain MXene sheets using milder conditions, preserving their size and reducing the occurrence of defective sites. In 2022, Shekhirev et al. reported a soft delamination method (stirring instead of shaking) to prevent the fracture of MXene sheets during the process, resulting in an extremely large lateral sheet size of >40 μm.^[^ ^70^ ^]^ Building on this modification, Thakur et al. developed an optimized and step‐by‐step methodology for etching and delaminating excess aluminum MAX phases in 2023 (Figure 13b), as referenced in Section 2.4.2. Besides enhanced oxidation stability, the MXene film produced using this optimized method exhibits electrical conductivity as high as ≈21 000 S cm^−1^. Moreover, the synthesis yield increased up to 38% (with respect to the mass of the starting MAX precursors), significantly higher than previously reported yields of ≈25–30%.^[^ ^16^ ^]^

The methods to tailor the surface of MXenes have been paramount in providing enhanced oxidation stability. Yoon et al. synthesized halogen‐free MXene using a NaOH‐based etching solution (Figure 13c).^[^ ^71^ ^]^ The halogen‐free MXene is unexpectedly stable against oxidation, as evidenced by changes in the UV–vis absorbance spectra of the samples incubated at 37 °C for over 4 weeks. The results showed that MXene produced from concentrated NaOH etching was much more resistant to oxidation compared to those produced from the minimally intensive layer delamination (MILD) method, despite having oxygen‐rich and fluorine‐free surface terminations. In a recent work by Zhang et al. a simple and efficient method for the delamination of MXenes derived from the molten salt synthesis technique was developed. LiCl salt and anhydrous polar organic solvents were used for delamination, resulting in Cl‐terminated single‐layer MXenes.^[^ ^72^ ^]^ Films produced from this MXene were flexible and had an electrical conductivity of 8000 S cm^−1^ that was retained after a week of exposure to 95% humidity, demonstrating successful delamination, preservation of inherent surface properties, and stability under high‐humidity conditions.

#### 2.4.4 Additional Studies on Capping Agents

Discussions in both Sections 2.2.1 and 2.3.3 highlighted the role of defects in the oxidation of MXenes and how capping agents can be utilized to protect these defects from oxidative attacks, whether against oxygen or water. Additional studies further expanded on testing a wider variety of capping agents. In 2019, Habib et al. assessed the oxidation of MXene/polyvinyl alcohol blends in water and organic solvent (under RT and freezing conditions) compared to pure MXene dispersions using conductivity measurements.^[^ ^73^ ^]^ The slowest decrease in conductivity was observed for all frozen samples, as well as the polymer composites at RT. Meanwhile, Zhang et al. reported in 2021 the advantage of tartaric acid as an additive for MXene/poly(3,4‐ethylenedioxythiophene) polystyrene sulfonate composites (MXene/PEDOT:PSS). The addition of tartaric acid protected MXene composites from oxidation, even at 60 °C and in water (Figure 13d). This was attributed to the coordination‐mediated capping effect of tartaric acid on the Ti cations in defective edge sites, consequently inhibiting oxidation.^[^ ^74^ ^]^ In 2022, Huang et al.^[^ ^45^ ^]^ investigated the effect of polyphosphate (PP) to the overall chemical stability of Ti_3_C_2_T_*x*_ MXene in aqueous solution by monitoring the evolution of methane in solutions with varied MXene:polyphosphate concentration ratios, including MXene dispersions exposed to both Ar and air. Using gas chromatography with a thermal conductivity detector (GC‐TCD) complemented by Raman spectroscopy, the rate of CH_4_ was found to be the least for the MXene‐PP solutions, which equates to the least degradation rate among the samples.

Surface functionalization of MXene with imidazolium salt ionic liquids (ILs) has also been shown to produce oxidation‐resistant MXene sheets. In a study by Zhao et al. in 2021, MXene nanosheets modified with 1‐(3‐aminopropyl)‐3‐methylimidazolium bromide demonstrated enhanced stability in air, retaining their 2D sheet‐like structures for 80 days.^[^ ^75^ ^]^ In a more recent study by Ning et al. in 2023, IL salts such as 1‐allyl‐3‐methylimidazolium bromide ([BMIM]^+^Br^−^) were found to protect MXene from oxidation when used as an intercalant and stabilizer during exfoliation.^[^ ^76^ ^]^ Interestingly, the authors of this work proposed that electrons originating from anatase by‐products react with dissolved oxygen to form anion free‐radical oxygen (•O_2_^−^), which then generates highly oxidizing hydroxyl radicals (•OH).^[^ ^62^ ^]^ The imidazolium ions electrostatically adsorbed on the surface of MXene inhibit the attack of these destructive •OH radicals, thereby protecting MXene from oxidation.Despite reports of MXene generating radicals during photochemical reactions^[^ ^61^ ^]^ and degrading faster under light exposure,^[^ ^14^ ^]^ a specific investigation into the impact of these reactive species on the oxidation process has not yet been conducted.

Recently, Tan et al. used Tris‐HCl buffer to stabilize a dilute MXene dispersion (0.05 mg mL^−1^).^[^ ^77^ ^]^ Tris‐HCl functionalized MXene maintained its original morphology, structure, and favorable dispersity even after 150 days of aging under naturally aerated conditions. Spectral analysis and multiscale simulations revealed that the combined pH regulation and defect/edge‐capping effects on MXene sheets prevented interaction with H_2_O and dissolved O_2_.

## 3 Inconsistencies in Oxidation Studies Leading to Discrepancies in Proposed Mechanism

Section 2 reviewed theoretical and experimental evidence highlighting the significant roles of both dissolved oxygen and water in MXene degradation within aqueous dispersions. Various factors, including flake size, defects, MAX phase quality, and storage conditions, were identified to influence the oxidation rate. While early studies indicated that MXene reacts directly with water,^[^ ^22^ ^]^ succeeding mitigation strategies have focused on minimizing MXene's contact with oxygen. Later, it was found that water degrades MXene much faster than atmospheric O_2_.^[^ ^44^ ^]^ The proposed mechanism of how water molecules attack the basal plane of MXene was first illustrated in 2022 using MD simulations.^[^ ^53^ ^]^ Despite efforts to explore the oxidation kinetics of MXene, fundamental studies remain scarce, and the precise mechanism continues to be debated.^[^ ^42^ ^]^ In this section, we offer our perspective on why discrepancies in the oxidation mechanism of MXenes persist to this day.

### 3.1 Lack of Conclusive Evidence on Hydrolysis during Early Studies

The lack of experimental evidence supporting MXene's oxidative hydrolysis initially led researchers to consider dissolved oxygen as the primary driving factor for the reaction. It was not until 2019 that Huang et al. conducted a systematic study on MXene oxidation, which aims to study the degradation kinetics of MXene in water‐rich and O_2_‐rich conditions independently to isolate and compare the influence of each agent to the overall rate. Findings show that after 30 days of exposure, much more extensive oxidation was observed in MXene dispersed in argon‐purged water, as evidenced by the decrease in Ti_3_C_2_T_*x*_ and increase in TiO_2_ from both UV–vis and Raman spectroscopic data, in contrast to that of the O_2_‐purged isopropyl alcohol, which showed no observable changes. In contrast, introducing other solvents can reduce the influence of water to MXene and hinder oxidation, as later demonstrated by Zhang et al.^[^ ^78^ ^]^ through the use of ethylene glycol (EG) and dimethylsulfoxide (DMSO) with water in formulating MXene‐based inks. However, it was also observed that MXene dispersions began aggregating beyond 60 vol% EG in water, suggesting that an optimal water‐solvent formulation should be carefully considered, which balances the compromise between oxidation stability and processability. The studies show how water primarily influences MXene oxidation, and that the mere presence of organic solvent reduced the oxidation rate. Whether this may be due to the competing interaction between the organic solvent and water with the MXene surface, or merely due to the overall reduction in the rate of water attack is still yet to be explored.

To understand the specific mechanism behind water‐induced oxidation, Wu et al. performed a FPMD simulation of the MXene‐water interaction, using monoterminated Ti_3_C_2_O_2_ MXene at RT.^[^ ^53^ ^]^ Results show how water approaches the MXene surface through strong hydrogen bonding with the O atoms at the T layer and H atoms of water, followed by the nucleophilic (electron‐donating) attack of the electron‐rich water O atom on the electron‐deficient Ti atom beneath the MXene T layer. An interesting observation is the apparent “pullout” of a surface Ti atom due to the nucleophilic attack, causing an irreversible bonding of Ti to the O atom of the adsorbed water. Although these results are only explicit to the monoterminated, pure oxygen‐terminated MXene, insights on the mechanism can still be applied to experimentally produced MXenes, since the predominant surface groups also contain oxygen (—O—, =O, OH). However, other mechanisms of nucleophilic water attack may arise from sites containing halogens (—F, —Cl), such as possible substitution by water or spontaneous release and exposure of the bare Ti surface defect, among others. The mechanism and contribution of such processes to the overall oxidation rate is an important fundamental direction that is yet to be explored.

### 3.2 Opposing Observations on Oxidation under Acidic and Basic Conditions

Studies have demonstrated that the oxidation stability of MXene is influenced by the pH of the dispersion, but recent experimental results have led to conflicting viewpoints. In 2020, Zhao et al. observed the accelerated oxidation of MXene in basic environments, attributing it to the instability of the deprotonated oxide anion (–O^−^).^[^ ^60^ ^]^ More specifically, the high pH environment equates to a larger concentration of hydroxide (^−^OH) anions, which can associate with the positively‐charged edges and defects, as established in antioxidant and polyanion capping studies^[^ ^39^, ^57^, ^65^ ^]^ mentioned in previous sections. The associated ^−^OH groups then deprotonate the –OH termination groups, which then produce the oxide (–O^−^), which are suspected to accelerate the oxidation process, although there are no further exploration nor proposals made regarding the mechanism. However, Doo et al. performed a systematic study resulting in an apparently opposite conclusion. In this study, the effect of both pH and temperature on the rate of oxidation of aqueous MXene dispersions is studied, and the relative oxidation rates are calculated based on the changes in the absorbance of MXene at different conditions. It was proposed that at low pH values, the excess H^+^ attacks the electronegative O atoms at the T layer of MXene, causing a localization of the electron density at the newly‐formed Ti—O bond. This localization results in increased exposure of the surface Ti atoms, increasing the susceptibility toward a nucleophilic attack from the O atoms of either H_2_O or dissolved O_2_, which promotes oxidation to Ti(IV). In contrast, the formed hydroxyl anion (–O^−^) at high pH values is stabilized through the association of the cations from the hydroxide salt (K^+^, Na^+^) introduced in the solution, resulting in increased stability of MXene dispersions toward oxidation.^[^ ^38^ ^]^ The hypotheses were supported by the significantly higher calculated activation energies of oxidation at higher pH values, especially at 20 and 40 °C. This conclusion was further supported by the work of Huang et al. in 2022,^[^ ^46^ ^]^ who found that a combination of high pH conditions and the addition of sodium ascorbate slows down the degradation of MXene, explaining that the combination of these conditions protects the MXene nanosheets from both oxidation and hydrolysis.

Looking closely at the studies, there are factors affecting the dispersions apart from the pH that were not monitored, leading to an apparently contrasting set of findings. For instance, it is also observed by Huang et al.^[^ ^46^ ^]^ that MXene sheets tend to coagulate and form multilayer structures in acidic conditions, limiting the interaction of water with the nanosheets. This knowledge, in fact, has been applied in the production of strong wet‐spun fibers.^[^ ^25^ ^]^ However, it was also found that the aggregates after acid treatment were floating in solution instead of precipitating at the bottom, possibly due to the presence of gaseous degradation products forming between the nanosheets despite the aggregation.

In contrast, basic conditions enhance single‐layer MXene dispersion, which maximizes the interaction with water and possibly oxygen, which may also enhance conditions that favor degradation. Apart from the dispersion behavior, the size distribution of MXene dispersions should also be monitored. Variations in both the size and dispersion behavior result in differences in the overall solvent‐accessible surface area, significantly impacting the observed degradation rate. In conclusion, the apparently conflicting conclusions on MXene stability at different pH may stem from factors that are outside its surface chemical reactivity.

### 3.3 Different Hypotheses on Oxidation Mechanisms at Different Sites of Ti_3_C_2_T_*x*_ MXene

Is MXene oxidation faster at the edges of the flake or the defective sites of the basal plane? This question is still currently debated due to differences in the mechanisms offered by different researchers at the initial stages of MXene oxidation. Early observations suggest that since the edges are more exposed to the immediate environment, it is also where oxidation starts before progressing to the basal plane.^[^ ^50^ ^]^ In contrast, Xia et al. showed how atomic defects on the basal plane serve as nucleation sites for oxidation, using atomic‐resolution scanning transmission electron microscopy (STEM).^[^ ^37^ ^]^ Specifically, it was observed that oxidation of Ti atoms generates Ti cations near the atomic defects and edges, while C atoms undergo oxidation at the Ti vacancies, leading to the formation of amorphous carbon. The presence of Ti vacancies facilitates the flow of electrons, creating an internal field at the defective sites. The work of Wu et al. also illustrated through FPMD simulations how the rich presence of H‐bonding sites at the basal plane attracts water molecules, and how these adsorbed water molecules can pull Ti atoms out from beneath the T layer and break the Ti—C bond, initializing the degradation process.^[^ ^53^ ^]^

In a separate study conducted by Kramer et al. in 2023, APT observations unveiled how oxide formation begins in the presence of alkali metals (Li, Na), halogens (Cl, F) as surface terminations, intercalated ions, or impurities, all introduced during the wet chemical synthesis of MXene.^[^ ^25^, ^66^ ^]^ It was further suggested that during wet chemical synthesis, preexisting pinhole defects may have created individual local electric fields that drove the flow of electrons and cationic Ti atoms, initiating the oxidation process. Further analysis of the initial degradation sites by Liu et al.^[^ ^67^ ^]^ revealed that the exposed (010) and (100) crystal planes of MXene exhibit faster oxidation than the (001) plane.

### 3.4 What Is Produced after Oxidation?

Understanding which by‐products form can provide further clarity on the kinetics of MXene oxidation. It is known that oxidative degradation of MXene results in the formation of anatase, but disagreements regarding other by‐products persist. Huang et al. proposed the formation of CH_4_.^[^ ^36^ ^]^ Doo et al. In contrast, concluded that the presence of dissolved CO_2_, H_2_CO_3_, and HF, aside from CH_4_, accounts for the decrease in pH upon oxidation.^[^ ^38^ ^]^ These gaseous species were detected using Gas Chromatography ‐ Time‐of‐Flight ‐ Mass Spectrometry (GC‐TOF‐MS); however, they were not considered in the reaction mechanism. Some of these gases are reactive and can possibly participate in the oxidation mechanism, which may accelerate the degradation of MXene. For example, the presence of dissolved CO_2_ and HF by‐products in the aqueous medium can cause a drop in pH, which may then acid‐catalyze the oxidation process, resulting in faster degradation.

### 3.5 Was the Impact of Oxidative Radicals Disregarded?

Under UV light, radicals are generated in the dispersion medium, which could potentially attack the MXene surface and induce further oxidation.^[^ ^61^ ^]^ It has been suggested that oxygen and moisture in the environment are transformed into radicals when UV light interacts with the anatase regions in MXene.^[^ ^61^, ^62^, ^63^ ^]^ Longer exposure to UV light produces more radicals, consequently leading to increased oxidation. Although most storage methods for MXene are already carried out in light‐free conditions, accounting for this process might alter existing oxidation mechanisms (regardless of whether oxygen or water is the driving species). However, further experiments are required to accurately evaluate the effects of UV radiation on MXene oxidation, specifically determining which radical species are produced.

### 3.6 Uncertainty on the Role of Additives

Discussions in Section 2.2.1 and 2.4.4 suggest incorporating polyanionic salts,^[^ ^57^ ^]^ citric acid,^[^ ^60^ ^]^ and other antioxidants^[^ ^39^ ^]^ into MXene to reduce oxidation. However, the precise mechanism by which these compounds mitigate oxidation remains unclear, and there is a lack of definitive evidence to support the proposed mechanisms. These studies raise questions such as: 1) do these compounds directly interact with MXene or with water? 2) Does MXene retain its properties after the addition of these compounds? and 3) Is the process reversible, allowing for easy removal from the MXene dispersion?

Furthermore, the exact function of additives in the oxidation process, particularly whether they scavenge oxygen or protect MXene to impede water access, remains somewhat uncertain. Efforts to clarify this confusion include the work of Zhao et al. in 2022 on the role of antioxidants.^[^ ^39^ ^]^ Initially, these compounds were employed to shield MXene from oxygen, but recent studies now indicate that they also prevent contact with water. Although the follow‐up study was conducted years after their initial research in 2019,^[^ ^41^ ^]^ it is recommended that the community make an effort to reference both studies, to clearly understand the progression in resolving this issue.

### 3.7 MAX Phases and MXenes Used in Early Oxidation Studies Were Suboptimal

Early investigations into MXene oxidation (pre‐2017) were conducted before the development of the optimized MILD method. Consequently, these findings may only apply to MXenes produced using HF, which are known to possess more F‐terminations than MILD and are considered more unstable and prone to oxidation.^[^ ^18^ ^]^ Similarly, protocols for MXene synthesis were not optimized at the time, resulting in inconsistent purity and properties of these MXenes. The contaminants found in MXene, including Li, Al, Na, and some halides, have the potential to alter the MXene surface and can either facilitate or inhibit oxidation.

Although recent studies have utilized optimized versions of MILD and mixed acid (HCl/HF) methods, most MXenes prior to 2022 were derived from MAX phases known to contain oxycarbides. While the excess metal method for synthesizing MAX phases was developed in 2021,^[^ ^12^ ^]^ it was only in 2022 that Michalowski et al. confirmed that most MXenes used in previous years did indeed contain oxycarbides,^[^ ^69^ ^]^ oxygen‐containing phases that are detrimental to their stability toward oxidation. This study was crucial as it had the potential to significantly revise nearly all hypotheses regarding the source or origin of oxidized species within the MXene structure. Furthermore, the optimized synthesis method for this type of MAX phase was reported by Thakur et al. only in 2023.^[^ ^16^ ^]^

## 4 Outlook on Reconciling Inconsistencies in the Literature and Further Understanding the Degradation Mechanism

The oxidation of MXene presents a significant challenge that requires immediate attention to enable the progress of MXenes and their derivatives for various applications. However, the obstacles highlighted in the preceding sections regarding the comprehension of the actual oxidation mechanism present a substantial barrier to developing an optimized approach for preserving MXene dispersions. In this section, future research directions are outlined with the intention of investigating strategies, additional crucial factors, and parameters to further improve our understanding of MXene oxidation.

### 4.1 A Comprehensive Study Encompassing All Known Critical Factors

To address the inconsistencies in the literature discussed in Section 3, a comprehensive study is needed. This study should assess all currently known factors that affect oxidation. It should also utilize recent methods for MAX and MXene synthesis, as much of the early work relied on materials derived from unoptimized methods. A methodical investigation that considers the impact of different surface terminations in MXenes may yield more conclusive results compared to studying MXenes with variable surface characteristics. Additionally, other factors known to influence the properties of MXene, such as the impact of agitation or shaking on sheet size, must be included. These factors should then be correlated with the current understanding of the mechanism. For example, researchers should investigate whether smaller flakes resulting from excessive agitation are more susceptible to oxidation.

### 4.2 Accounting the Influence of By‐Products

Studying the influence of by‐products (e.g., gaseous products, radicals, acids) provides another avenue for advancing our understanding of MXene oxidation kinetics. In addition to discrepancies in the reported oxidation products and their absence from previously proposed mechanisms, these by‐products themselves may be reactive and potentially catalyze MXene oxidation, akin to a self‐catalyzing oxidation process. For example, the interaction of UV radiation with anatase (an initial by‐product produced during MXene oxidation) may lead to the generation of O· and OH· radicals in the dispersing media, a phenomenon overlooked in recent oxidation studies.

### 4.3 Development of Experimental Methods to Further Improve Knowledge on MXene Surface, Structure, and Oxidation Behavior

A long‐standing limitation of MXene oxidation research is the lack of available experimental characterization methods needed to observe the surface and edge chemistry of MXenes. To date, much of our understanding of the surface and edge structures of MXenes relies on results derived from simulation experiments, typically conducted to validate the theoretical viability of experimental observations. Experimental efforts aimed at observing these structures could either substantiate or disprove the myriad theoretical and computational research findings, thereby shedding light on the actual properties of MXene and its behavior toward oxidizing agents.

Several attempts have been made to map MXene surface components through advanced nuclear magnetic resonance spectroscopy,^[^ ^18^, ^79^ ^]^ Raman spectroscopy,^[^ ^80^, ^81^ ^]^ and X‐ray photoelectron spectroscopy (XPS)^[^ ^79^, ^82^, ^83^, ^84^ ^]^ analysis. However, each of these techniques has its limitations. For example, elemental analysis methods like SEM or TEM‐assisted energy‐dispersive X‐ray analysis,^[^ ^85^, ^86^ ^]^ can determine the amount of oxygen atoms on the MXene surface and edge structures, but identifying specific species (—OH, =O, —O—, —O^−^, —Ox) remains challenging. XPS offers the potential for such characterization, although only qualitatively, especially considering the variabilities in the morphology of sampled MXenes, such as size polydispersity and the presence of multilayered structures.

Drawing upon the understanding of MXene surface and edge structures, several experiments can be developed to monitor both its oxidation dynamics and kinetics further. Conducting in situ experiments represents a logical next step in elucidating this process. To comprehend the oxidation mechanism at the atomic level, a more thorough investigation is essential. This entails observing and measuring directly within the reaction environment where MXene oxidation occurs. Such experiments facilitate real‐time tracking and analysis of the chemical transformations and structural changes undergone by MXene during oxidation.

Techniques such as in situ XRD, APT, GC, Fourier transform infrared, Raman, XPS, and TEM can be employed individually or in‐tandem to gather detailed information about oxidation kinetics, intermediate species formation, and structural evolution of MXene under various oxidation conditions.

### 4.4 Computational Methods to Understand the Influence of Competing/Synergistic Factors

**Figure 14:**
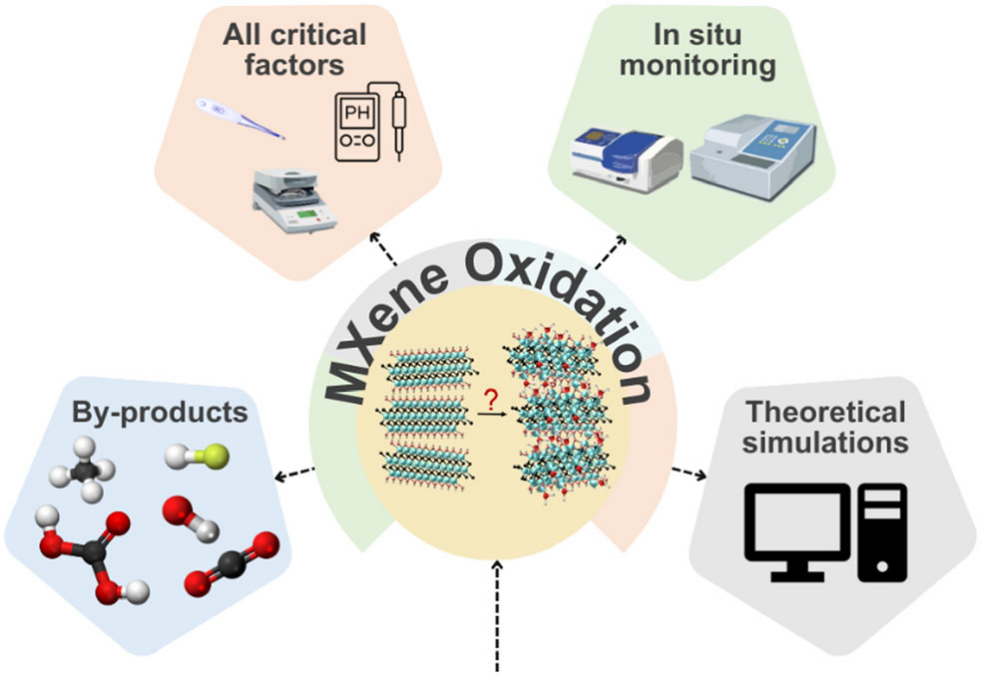
Outlook on how to further understand MXene oxidation.

Establishing a definitive correlation between theoretical simulations and the data gathered from different variables in real experiments is also crucial for deciphering the actual degradation mechanism (**Figure** **14**). Computational techniques like MD simulations can offer insights into understanding how various factors interact, potentially competing or mutually reinforcing each other's effects. Despite the availability of experimental studies on the individual effects of these factors, it is essential to consider the confounding effects, especially in designing long‐term stability experiments. For example, this approach could be beneficial in understanding the unexplored potential of selective interactions between MXene and organic solvent molecules, hindering oxygen attack on the oxidizable parts of MXene (as experimentally observed by Huang et al. in 2019). Additionally, it could shed light on the reasons behind the preservation of MXene in organic solvents, even in the presence of water, as observed by Zhang et al. in 2021. Computational simulations can also assist in elucidating the true function of additives, determining whether they obstruct MXene's contact with water or hinder oxidation by quenching reactive species such as oxygen and radicals.

## 5 Summary and Conclusion

The oxidative degradation of MXene stands as a primary challenge demanding immediate attention to enable further development of MXene dispersions and their corresponding architectures for various applications. While the community has demonstrated considerable effort in addressing this crucial issue, conflicting perspectives on the actual oxidation mechanism of MXene have emerged, presenting hindrances in devising comprehensive preservation methods for these dispersions.

Summarizing the key discoveries through this review has highlighted those inconsistencies primarily arose due to the common lack of definitive evidence in initial studies, compounded by subsequent investigations founded on assumptions derived from these early inconclusive findings. With the rapid growth of the field, the urgency to generate follow‐up oxidation studies may have influenced the methods intended for collecting definitive evidence.

Addressing this issue necessitates a thorough investigation that considers all presently recognized factors influencing oxidation and employs the latest techniques for MAX and MXene synthesis. Many early studies relied on materials derived from dated synthesis protocols; however, it is recognized that MXene synthesis and processing methods are dynamic and constantly evolving. It is vital to encourage the scientific community to delve into MXene oxidation both experimentally and computationally to gather a broader array of information that could eventually aid in understanding this complex process.

Confidently, MXenes will persist as the foundation for developing next‐generation devices. Shedding light on the mysteries of MXene oxidation will further harness their potential in producing innovative structures and properties in nanoscale and macroscale assemblies. Insights acquired from this review can also be extended to other nanomaterials that typically undergo solution processing and are also susceptible to oxidative degradation (e.g., black phosphorus, borophene), potentially catalyzing new breakthroughs that will advance the wider field of materials science and engineering.

## Conflict of Interest

The authors declare no conflict of interest.
